# CD2 augmentation enhances CAR-T-cell efficacy via immunological synapse remodeling and T-cell exhaustion mitigation

**DOI:** 10.1038/s41423-025-01314-6

**Published:** 2025-07-04

**Authors:** Qi Zhu, Jiajia Li, Nan Liu, Lu Han, Zhiqiang Wu, Yao Wang, Xin Lin, Jianshu Wei, Weidong Han

**Affiliations:** 1 Department of Biotherapy, the First Medical Center, Chinese PLA General Hospital, Beijing, China; 2 Changping Laboratory, Beijing, China; 3 School of Basic Medical Sciences, Tsinghua University School of Medicine, Beijing, China; 4 Tsinghua-Peking Center for Life Sciences, Beijing, China

**Keywords:** CAR-T cells, CD2, immunological synapse, T cell exhaustion, Antigen sensitivity, Immunoediting, Cancer immunotherapy

## Abstract

CAR-T-cell therapy has made significant strides in treating hematological malignancies, yet its efficacy is often hampered by suboptimal T-cell functionality, marked by weak antitumor capabilities and a lack of durability. The immunological synapse, a key determinant of T-cell function, is influenced by the CD58-CD2 axis. The dynamic regulation of CD2 expression on T cells impacts the quality of CAR-mediated immunological synapses, affecting CAR-T-cell functional outcomes and differentiation. Our study demonstrated that CD2 expression levels are closely linked to the quality of immunological synapses formed by CAR-T cells and their antitumor potency. Exogenous CD2 supplementation enhances the ability of CAR-T cells to form high-quality synapses, reduces T-cell exhaustion, and increases sustained antitumor efficacy. Additionally, ectopic CD2 expression increases CAR-T-cell sensitivity to low-density antigens. Thus, replenishing CD2 in CAR-T cells is a promising strategy to increase the therapeutic efficacy of CAR-T-cell therapy.

## Introduction

Chimeric antigen receptor T (CAR-T) cell therapy has achieved remarkable success in the treatment of hematologic malignancies [1–4]. Despite impressive remission rates, approximately half of patients relapse within a year after treatment [5–7]. The inadequate persistence of CAR-T cells and the loss of target antigens in tumor cells are the two primary factors that impede long-term therapeutic benefits [8–10]. Various strategies have been explored to address these challenges, including refining CAR molecules [11, 12], targeting more tumor-associated antigens [13, 14], modulating the epigenetic state of T cells [15, 16] and manipulating signaling pathways [17, 18]. However, the efficacy of these strategies is still limited by the complexities of real-world clinical scenarios. Therefore, there is an ongoing need to delve deeper into the mechanisms that regulate CAR-T-cell function to fully unleash the potential of CAR-T-cell therapy, with a particular emphasis on strategies that exhibit universal applicability.

The formation of immunological synapses (ISs) is a prerequisite for T-cell-mediated antitumor responses, orchestrating a chain of subsequent events such as cytoskeletal reorganization in T cells, directed release of cytotoxic granules [19], and activation of downstream signaling pathways [20, 21] that are instrumental in modulating T-cell expansion [22], metabolic activity [23] and lineage commitment [24, 25]. Thus, optimizing the IS has been widely regarded as a critical strategy to increase the efficacy of CAR-T-cell therapies.

The IS is a complex structure that relies on the precise and coordinated engagement of various ligand‒receptor pairs, such as ICAM1‒LFA1 [26], MHC‒CD4‒CD8 [27, 28], CD45 [29], and the CD58‒CD2 axis [30], which are highlighted in this research. In contrast to T-cell receptor (TCR)-mediated IS, CAR-mediated IS has diminished structural integrity, potentially accounting for the inferior cytotoxic persistence, lower antigen sensitivity and increased susceptibility to exhaustion observed in CAR-T cells [31, 32]. This finding also highlights the molecular disparities between the TCR and CAR systems [33].

The CD58-CD2 axis has been established as crucial for the formation of high-quality ISs within the TCR system [30]. In the CAR system, previous studies have also demonstrated that the absence of CD58 on tumor cells compromises IS integrity [34]. Unlike the specific case where a lack of CD58 leads to a total loss of CD58-CD2 axis function, in more common scenarios where tumor cells express CD58, the dynamic variation in CD2 expression levels on T cells [35] becomes the primary determinant affecting the axis’s function. This finding prompted us to suspect that suboptimal CD2 expression on T cells might hinder their ability to form high-quality ISs, thereby affecting the antitumor efficacy and lineage differentiation of T cells. This assumption is also informed by other clues, such as the diminished CD2 expression observed in terminally differentiated T cells [36, 37].

In this study, we noted that CD2 expression was promptly upregulated upon T-cell activation. However, as T cells differentiate toward a terminal state, T cells gradually lose their ability to upregulate CD2 in response to antigenic stimulation. The ectopic expression of CD2, which sustains elevated CD2 levels, was shown to optimize CAR-IS, alleviate T-cell exhaustion, and augment the antitumor potential of CAR-T cells. In conclusion, CD2 supplementation may serve as an effective strategy to increase the antitumor efficacy of CAR-T cells by optimizing the IS.

## Results

### CD2 is closely associated with T-cell function

**Fig. 1:**
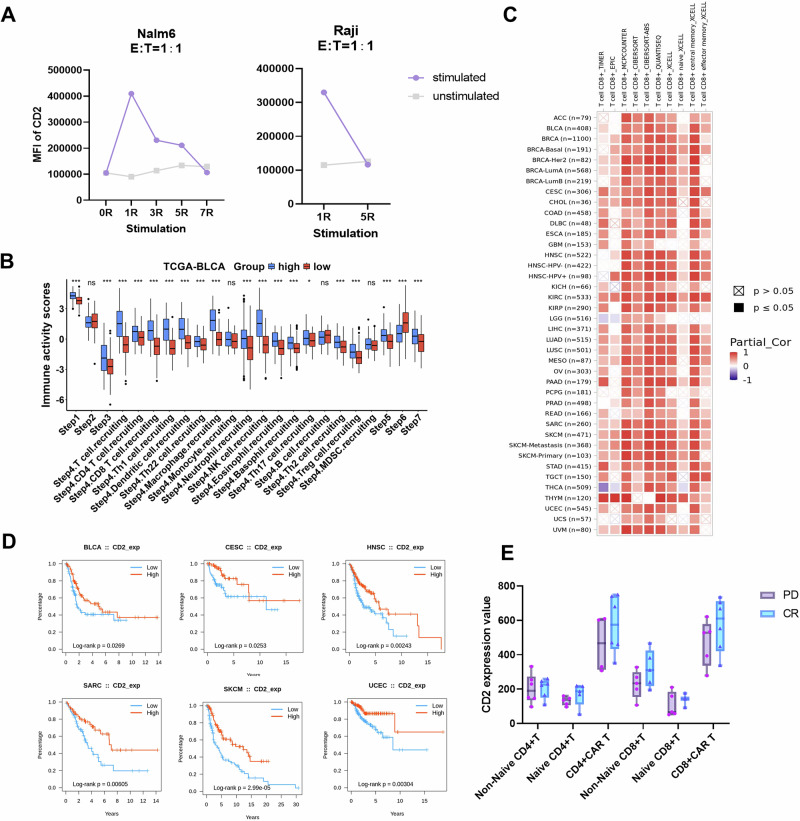
The expression level of CD2 is an indicator of T-cell function. **A** CD2 expression levels in CAR-T cells were measured via flow cytometry after chronic stimulation with Nalm6 cells or Raji cells at the indicated effector:target (*E*:*T*) ratios (*n*  = 3; mean ± SEM). **B** Association between CD2 expression and cancer-immunity cycle activity in the TCGA-BLCA cohort (data from TIP). Patients were stratified into high (blue) and low (red) CD2 expression groups on the basis of median CD2 expression levels. Statistical analysis, including 17 detailed steps of the cancer-immunity cycle, was performed to compare immune activity between these two groups. The Mann‒Whitney *U* test was used to assess differences. **C** Correlations between the CD2 expression level and CD8^+^ T-cell immune infiltration in cancers (data from TIMER2). The heatmap presents the adjusted Spearman rank correlation coefficients in various cancer types. **D** Associations between CD2 expression and overall survival across specific human cancers (data from TISIDB). **E** CD2 expression value in the cell population of initial apheresis T cells. CR complete response (*n* = 6); PD progressive disease (*n* = 6). Ns not significant, *p  <  0.05, **p <   0.01, ***p  <  0.001, ****p < 0.0001

Initially, we assessed the expression of CD2 on T cells across various activation states and differentiation stages to more precisely delineate its dynamic patterns. As shown in Fig. S1A, CD2 was expressed at low levels in resting T cells and was significantly upregulated upon activation, with expression levels gradually returning to baseline as the stimulus was withdrawn. Compared with naïve and central memory T cells, effector T cells presented greater CD2 expression (Fig. S1B), and CD8-positive T cells presented significantly greater CD2 expression than did CD4-positive T cells (Fig. S1C). By inducing exhaustion in CAR-T cells through multiple rounds of stimulation with tumor cells, we observed a progressive decrease in CD2 expression on T cells as exhaustion progressed (Fig. 1A).

Next, we employed database analysis to further delineate the correlation between CD2 expression and T-cell function. As shown in Figs. 1B and S2A, patients with higher CD2 expression presented significantly higher scores for immune cell priming/activation (Step 3) and recruitment of diverse T-cell subsets (Step 4), suggesting a link between CD2 expression and T-cell functionality. Additional analysis with TIMER (2.0) confirmed a positive correlation between CD2 expression levels and the abundance of tumor-infiltrating lymphocytes (TILs), as shown in Figs. 1C and S2B. Expanding our investigation, TISIDB database analysis revealed that across various tumor types, higher expression of CD2 was associated with a prolonged overall survival period and a reduced risk of mortality (Figs. 1D and S2C). We also investigated the association between CD2 expression levels and the therapeutic efficacy of CAR-T-cell therapy. Among a cohort of patients who received tandem CAR-T-cell therapy for diffuse large B-cell lymphoma, we noted that CD2 expression levels in those with treatment resistance were slightly lower than those in patients who achieved complete remission, although this difference did not reach statistical significance (Fig. 1E).

These results collectively underscore a positive association between CD2 expression levels and T-cell function.

### CD2 plays a decisive role in determining the quality of immune synapses

To confirm whether the difference in CD2 expression is an independent factor driving the differences in CAR-T-cell function and differentiation, we assessed the role of CD2 in CAR-mediated IS formation. This is key, as ISs are fundamental to the functional output and subsequent differentiation of T cells, with the CD58-CD2 axis known to regulate the assembly of TCR-mediated synapses.

**Fig. 2:**
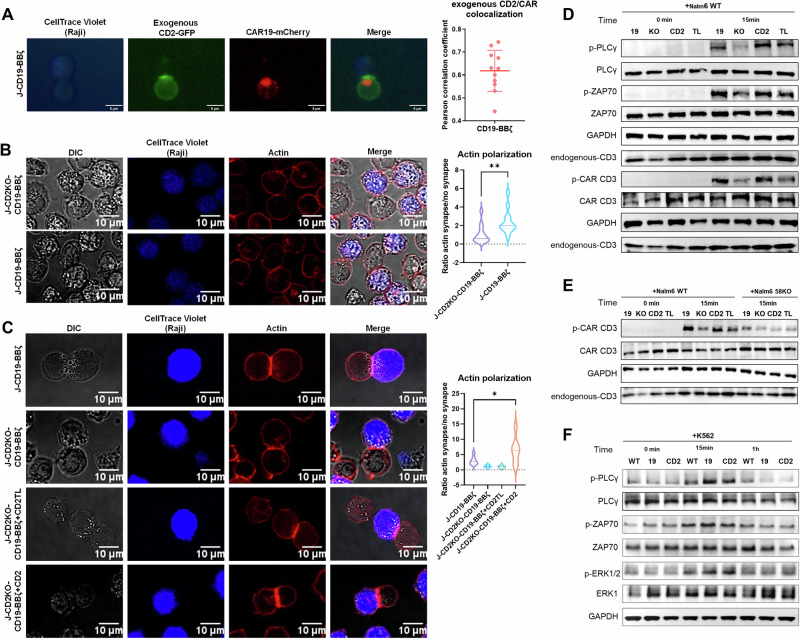
CD2 is decisive for the quality of CAR-IS. **A** Immunofluorescence imaging (left) and colocalization analysis (right) of CD2 (green) and CAR (red) in Jurkat CD19-BBζ CAR-T cells. The data are presented as Pearson’s correlation coefficient (PCC) values (*n* = 10; mean ± SEM; scale bar = 5 μm). **B** Confocal microscopy of Jurkat CAR-T cells in combination with Raji cells (left). Raji cells were labeled with CellTrace™ Violet dyes. Fixed and permeabilized cells were stained with F-actin (TRITC Phalloidin). The IS was evaluated by measuring the ratio of F-actin in and out of synapses (right) (*n*  =  30; mean ± SEM; scale bar = 10 μm; unpaired *t* test). **C** Confocal microscopy of Jurkat CAR-T cells (J-CD19-BBζ), Jurkat CD2 null CAR-T cells (J-CD2KO-CD19-BBζ), J-CD2KO-CD19-BBζ cells expressing the extracellular domain of CD2 (J-CD2KO-CD19-BBζ + CD2TL) and J-CD2KO-CD19-BBζ cells expressing full-length CD2 (J-CD2KO-CD19-BBζ + CD2) in conjugation with Raji cells. Raji cells were labeled with CellTrace™ Violet dyes. TRITC-phalloidin was used to stain F-actin. The IS was evaluated by measuring the ratio of F-actin in and out of synapses (right) (*n* = 30; mean ± SEM; scale bar = 10 μm; ordinary one-way ANOVA). **D** CAR ligation initiates proximal signaling events in J-CD19-BBζ (marked as 19), J-CD2KO-CD19-BBζ (marked as KO), J-CD2KO-CD19-BBζ + CD2 (marked as CD2) and J-CD2KO-CD19-BBζ + CD2TL (marked as TL) cells. **E** Proximal signaling events in Jurkat CAR-T cells upon stimulation with Nalm6 WT or Nalm6 CD58KO cells. **F** Activation signals of J-CD19-BBζ (marked as 19) and J-CD19-BBζ + CD2 (marked as CD2) CAR-T cells cocultured with CD19^−^CD58⁺ K562 cells. The image is uniformly brightness/contrast adjusted; see supplementary materials for raw data *p  <  0.05, **p   <   0.01

First, by coculturing Jurkat cells stably expressing CAR19 (J-CD19-BBζ) with Nalm6 tumor cells, we observed significant CD2 clustering in the region where CAR molecules aggregated (Fig. 2A), providing direct evidence of the involvement of CD2 in IS formation. Deletion of CD2 in J-CD19-BBζ cells led to substantial disruption of IS morphology, as evidenced by the impaired clustering of F-actin at the cell‒cell contact site (Fig. 2B). Correspondingly, the absence of CD58 on tumor cells also significantly hindered IS formation (Fig. S3).

Next, we complemented CD2-null J-CD19-BBζ cells with ectopic expression of full-length CD2 or its extracellular domain, and both constructs led to a substantial increase in CD2 expression, even surpassing that of wild-type Jurkat cells (Fig. S4A, B). As shown in Fig. 2C, the restoration of full-length CD2 significantly enhanced F-actin polarization at the IS site, whereas the extracellular domain had a negligible effect, suggesting that the intracellular domain of CD2 is essential for driving cytoskeletal rearrangements. Western blotting also revealed that the loss of CD2 significantly diminished the ability of the IS to trigger activation signals, which could be rescued by the restoration of CD2 (Fig. 2D). Notably, despite little improvement in IS morphology, overexpression of the extracellular domain alone also modestly enhanced activation signals, suggesting that CD2-CD58 binding can augment the capacity of the IS to initiate downstream activation signals via mechanisms beyond the direct activation of the CD2 signaling cascade. When CD58 was absent on Nalm6, reconstituting CD2 failed to augment T-cell activation signals (Fig. 2E). To investigate this further, we cocultured Jurkat-CAR-T-19 and Jurkat-CAR-T-19-CD2 cells with K562 cells, which lack the target antigen but express CD58. We found that overexpressing CD2 did not result in stronger activation signals (Fig. 2F). Moreover, immunofluorescence imaging revealed that overexpressing CD2 significantly enhanced the exclusion of CD45 from the immunological synapse (Fig. S4C). The above results demonstrated that CD2 is crucial for the morphology and function of the IS mediated by CAR molecules.

### Ectopic CD2 expression enhances the affinity between CAR-T cells and tumor cells

**Fig. 3:**
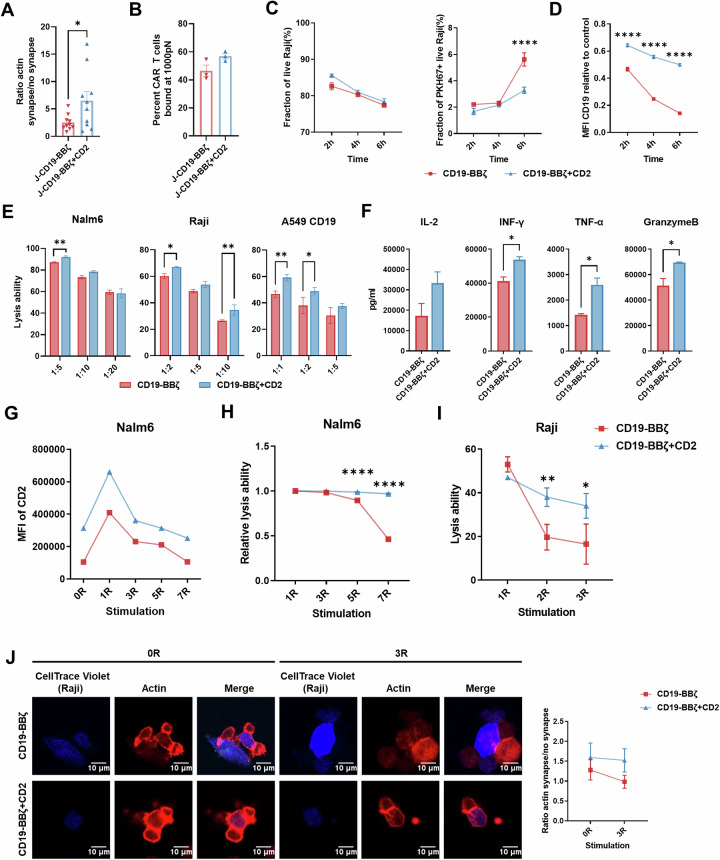
CD2-CAR-T cells demonstrate enhanced antitumor potential. **A** The quality of the IS formed by J-CD19-BBζ and J-CD19-BBζ + CD2 CAR-T cells conjugated with Nalm6 cells (*n* = 10; mean ± SEM; unpaired *t* test). **B** The percentages of J-CD19-BBζ and J-CD19-BBζ + CD2 CAR-T cells remaining bound to Nalm6 cells at 1000 pN (*n* = 3; mean ± SEM; unpaired *t* test). **C** The proportion of live Raji cells during the 6-h coculture with CAR-T cells at an *E*:*T* ratio of 1:10 (left). The proportion of PKH67+ Raji cells that contacted CAR-T cells among live Raji cells (right). **D** The expression level of CD19 on live Raji cells (*n* = 3; mean ± SEM; two-way ANOVA). **E** Lysis efficiency of Nalm6, Raji and A549-CD19 CAR-T cells treated with CD19-BBζ and CD19-BBζ + CD2 CAR-T cells at the indicated *E*:*T* ratios for 24 h (*n* = 3; mean ± SEM; two-way ANOVA). **F** Concentrations of cytokines released by CD19-BBζ- and CD19-BBζ + CD2 CAR-T cells after 24 h of coculture with Raji cells at an *E*:*T* ratio of 1:10. (*n* = 3; mean ± SEM; unpaired *t* test). **G** CD2 expression levels in CD19-BBζ and CD19-BBζ + CD2 CAR-T cells after each stimulation with Nalm6 cells at an *E*:*T* ratio of 1:1. (*n*  =   3; mean ± SEM). **H** Lysis efficiency of CD19-BBζ CAR-T cells and CD19-BBζ + CD2 CAR-T cells against Nalm6 cells at an *E*:*T* ratio of 1:1 for 24 h in each round of coculture (*n* = 3; mean ± SEM; two-way ANOVA). **I** Lysis efficiency of CD19-BBζ and CD19-BBζ + CD2 CAR-T cells against Raji cells at an *E*:*T* ratio of 1:5 for 24 h in each round of coculture (*n* = 3; mean ± SEM; two-way ANOVA). **J** Confocal microscopy of CD19-BBζ and CD19-BBζ + CD2 CAR-T cells (before and after three rounds of stimulation) stimulated with Raji cells. Raji cells were labeled with CellTrace™ Violet dyes. TRITC-phalloidin was used to stain F-actin. The IS was quantified by measuring the fluorescence density ratio of F-actin in and out of synapses (left) (*n* = 11, mean ± SEM; scale bar = 10 μm). *p < 0.05, **p < 0.01, ****p < 0.0001

On the basis of the aforementioned results, we intended to examine whether ectopic CD2 expression (Figure S5A–C) could further optimize the IS in the presence of endogenous CD2, which was gradually silenced alongside the occurrence of exhaustion. Initially, we observed that elevated CD2 expression refined the IS morphology, underscored by a pronounced increase in F-actin polarization within the IS region upon coculturing J-CD19-BBζ cells with Nalm6 cells (Fig. 3A).

Studies suggest that CAR-T-cell-mediated ISs are inherently unstable, resulting in frequent but fleeting contacts with tumor cells that do not effectively induce cytotoxicity, known as nonlytic contacts [38, 39]. To evaluate the effect of ectopic CD2 expression on CAR-T-cell–tumor cell interactions, we measured avidity via dynamic ultrasound microscopy (z-Movi) [40]. Our results revealed that CAR-T cells with elevated CD2 expression (J-CD19-BBζ + CD2) presented markedly greater avidity for Nalm6 cells than did their conventional counterparts (Fig. 3B). Additionally, cell adhesion assays demonstrated that epidermal growth factor receptor (EGFR)-BBζ + CD2 T cells adhered more robustly to A549 cells, a phenomenon not observed with CAR-T cells overexpressing only the extracellular domain of CD2 (Fig. S5D). To further elucidate the effects of CD2 overexpression on CAR-T-cell‒tumor cell interaction patterns, we labeled CAR-T cells with the membrane-incorporated dye PKH67, cocultured them with Raji cells, and monitored PKH67 transfer rates (Fig. S6A‒C). As shown in Fig. 3C, CD2 overexpression significantly decreased the transfer of PKH67 from CAR-T cells to Raji cells, indicating a reduced frequency of contacts. Studies have confirmed that CD19 on tumor cells is internalized upon contact with their corresponding CAR molecules. Consequently, the incidence of nonlytic contacts can be quantified by enumerating viable Raji cells exhibiting diminished CD19 expression following coculture with CAR-T cells [41]. Compared with conventional CAR-T cells, CD19-BBζ + CD2 CAR-T cells significantly mitigated the emergence of CD19-dim Raji cells, indicating that elevated CD2 expression effectively minimized nonlytic contacts (Figs. 3D and S6D).

Collectively, these findings suggest that supplementing CAR-T cells with exogenous CD2 could further optimize the morphology of the IS, increase its affinity for tumor cells, and diminish the frequency of contacts, notably those that are noncytotoxic.

### Ectopic CD2 expression enhances the antitumor potential of CAR-T cells

To determine whether the optimization of IS through CD2 overexpression could subsequently translate into enhanced antitumor efficacy, we assessed the tumor lysis ability of CAR-T cells across various systems. As shown in Fig. 3E, F, after 24 h of coculture, compared with their nonoverexpressing counterparts, CD19-BBζ + CD2 CAR-T cells presented a marginal yet significant cytotoxic advantage and increased cytokine secretion. This enhancement in cytolytic activity was similarly observed in another CAR-T system employing CD28 as the costimulatory domain (Fig. S7A, B).

A series of iterative cytotoxicity assays were subsequently conducted to assess the long-term antitumor efficacy of CAR-T cells. Notably, CD2 expression on CAR-T cells gradually decreased after repeated stimulation by tumor cells, whereas CD19-BBζ + CD2 CAR-T cells maintained significantly increased levels of CD2 expression (Fig. 3G). After multiple rounds of coculture, the tumor-killing ability of conventional CAR-T cells began to decline noticeably, in contrast to that of CD19-BBζ + CD2 CAR-T cells, which consistently displayed vigorous tumoricidal activity (Fig. 3H, I). Furthermore, with decreasing cytotoxicity and CD2 expression, the quality of the IS formed by conventional CAR-T cells significantly deteriorated, as evidenced by weakened F-actin polarization (Fig. 3J). In contrast, the ability of CD19-BBζ + CD2 CAR-T cells to form high-quality ISs was better preserved.

**Fig. 4:**
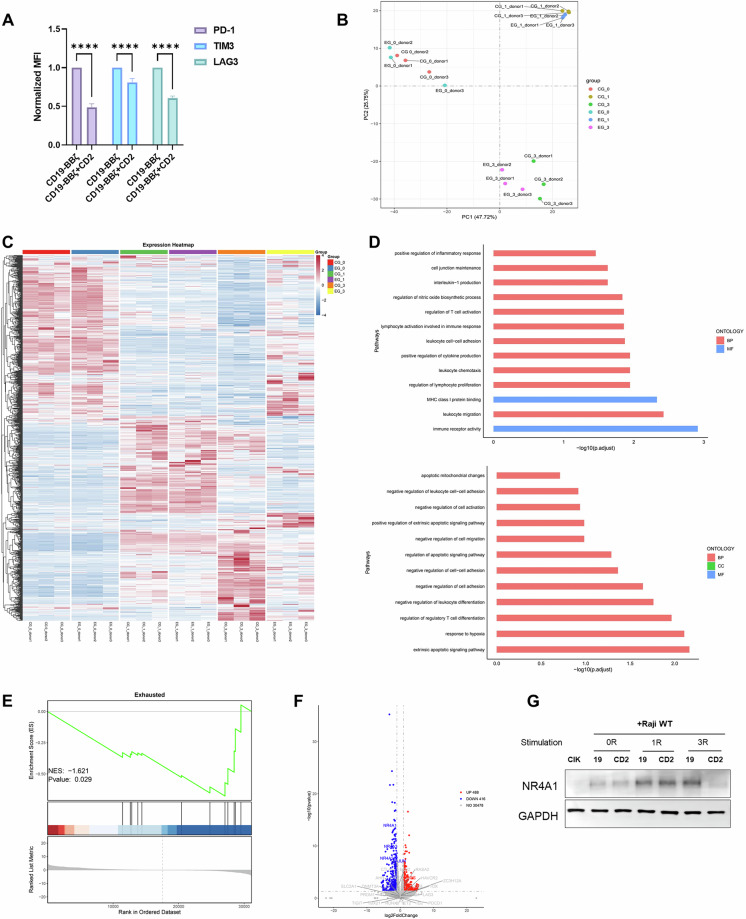
CD2-CAR-T cells exhibit divergent differentiation trajectories. **A** Mean fluorescence intensity (normalized to that of the control) of inhibitory markers on CD19-BBζ and CD19-BBζ + CD2 CAR-T cells following three rounds of stimulation with Raji cells at an *E*:*T* ratio of 1:5 (*n* = 3 donors; mean ± SEM; two-sided Wilcoxon test). **B** Principal component analysis (PCA) was conducted on RNA sequencing data derived from CD19-BBζ CAR-T cells (CG group) and CD19-BBζ + CD2 CAR-T cells (EG group). This analysis was performed both before and after one round, as well as three rounds of stimulation with Raji cells (*n* = 3 different PBMC donors per group). **C** Heatmap depicting the DEGs between the EG and CG. **D** Selected pathways derived from the gene ontology enrichment analysis of upregulated genes (top) and downregulated genes (bottom). **E** Representative GSEA plot showing the exhausted gene set of CAR-T cells after three rounds of costimulation. **F** Volcano plot illustrating the genes differentially expressed between CD19-BBζ + CD2 CAR-T cells and CD19-BBζ CAR-T cells after three rounds of stimulation with Raji cells. Transcripts that exhibit a false discovery rate (FDR) of less than 0.05 are highlighted in blue and red. **G** NR4A1 expression in CD19-BBζ (19) and CD19-BBζ + CD2 (CD2) CAR-T cells under acute (1R) or chronic (3R) antigen stimulation with Raji WT cells at an *E*:*T* ratio of 1:5. ****p < 0.0001

In addition to its direct impact on functional outputs, we next aimed to investigate whether the overexpression of CD2 influences the intrinsic properties of CAR-T cells. After 3 rounds of coculture with Raji cells, we examined the expression of T-cell dysfunction markers, which were considerably lower in the CD19-BBζ + CD2 CAR-T cells (Fig. 4A). Transcriptome sequencing was also performed on CAR-T cells that had undergone multiple rounds of coculture with Raji cells. The CD19-BBζ and CD19-BBζ + CD2 CAR-T cells showed minimal differences before and after the first round of antigen challenge. However, following three rounds of antigen stimulation, significant disparities in the two types of CAR-T cells were observed (Fig. 4B, C). After three rounds of antigen stimulation, the upregulated genes were enriched in pathways related to immune receptor activity, leukocyte migration, leukocyte cell–cell adhesion, regulation of T-cell activation, and maintenance of cellular junctions in CD19-BBζ + CD2 CAR-T cells. In contrast, the downregulated genes were enriched in the extrinsic apoptotic signaling pathway, negative regulation of leukocyte cell–cell adhesion, negative regulation of cell activation, and negative regulation of cell migration (Fig. 4D). Gene set enrichment analysis (GSEA) further confirmed that CD19-BBζ + CD2 CAR-T cells presented significantly reduced expression of exhaustion-related genes (Fig. 4E). In subsequent investigations, we meticulously examined the expression profiles of a set of genes that have been reported to significantly affect the antitumor efficacy of CAR-T cells, including *T-bet*, *Id2*, *Blimp-1*, *Runx3*, *AhR*, *TOX*, *NR4A*, *TCF7*, *BATF3*, *TET2*, *c-FOS*, *c-Jun*, *Dnmt3a*, *PDCD1*, *TIGIT*, *CD38*, *ENTPD1 (CD39)*, *CTLA4 (CTLA-4)*, *RASA2*, *EZH2*, *KLRG1*, *PD-1*, *CD28*, *CD27*, *TIM-3*, *LAG-3*, *GLUT1*, *Regnase-1* and *ITK*. As depicted in Fig. 4F, notable downregulation of NR4A1, NR4A2 and NR4A3 was coincidently observed in CD19-BBζ + CD2 CAR-T cells. As shown in Fig. 4G, all CAR-T-cell groups exhibited marked upregulation of NR4A1 expression following one round of coculture. Notably, after three coculture cycles, compared with conventional CAR-T cells, CD2-overexpressing CAR-T cells presented significantly lower NR4A1 expression levels. On the basis of the well-established role of NR4A in impairing mitochondrial fitness and sustaining the antitumor efficacy of CAR-T cells, omics analysis suggested that the overexpression of CD2 might reduce the differentiation of CAR-T cells toward an exhausted state by decreasing the expression of NR4A family proteins during repeated antigen stimulation.

### CD2-CAR-T cells demonstrate superior in vivo antitumor efficacy and proliferation potential

To evaluate the in vivo antitumor efficacy of CD19-BBζ + CD2 cells, we initially established an acute leukemia model by engrafting 5 × 10^5^ Nalm6 tumor cells overexpressing luciferase (Nalm6-L) one week prior to CAR-T-cell therapy. Following bioluminescence imaging (BLI) for tumor burden assessment and equalization, 3×10^5^ purified CAR-T cells (cultured for 12 days) were administered. As shown in Fig. S8A–D, while tumor growth was not effectively controlled in all treatment groups, possibly due to an overwhelming initial tumor burden, the group treated with CD19-BBζ + CD2 CAR-T cells presented the lowest tumor burden and a trend toward prolonged survival.

**Fig. 5:**
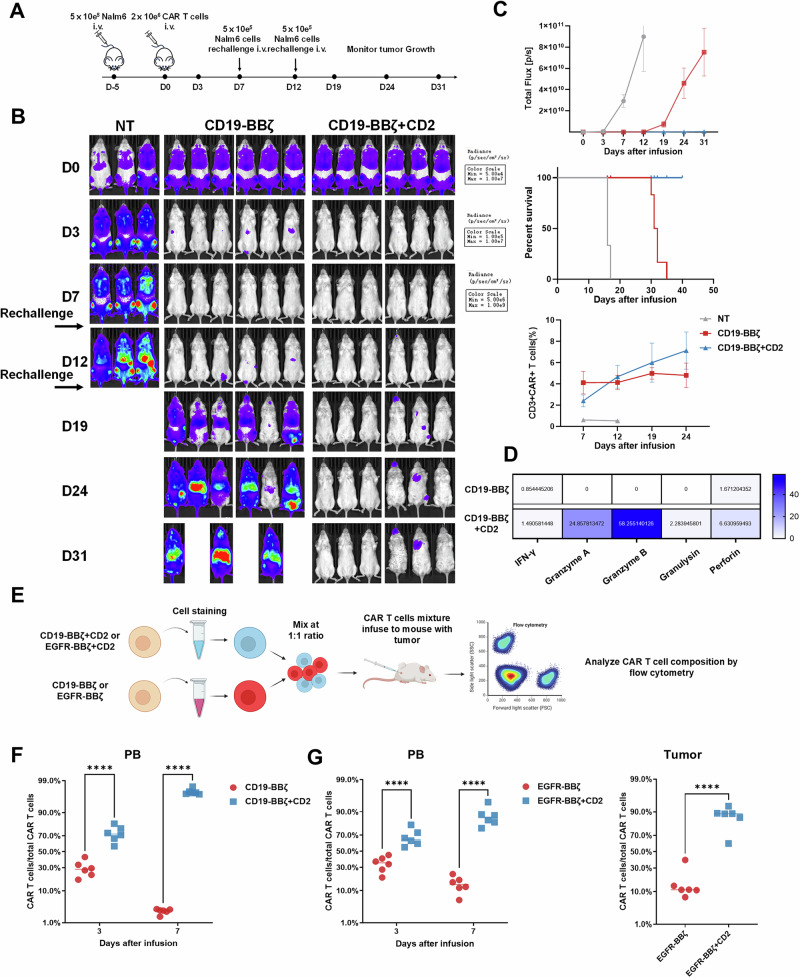
CD2-CAR-T cells outperformed conventional CAR-T cells in xenograft tumor models. **A** Schematic showing the generation of multiple challenge xenograft mouse models. BLI was performed at the indicated time points. **B** BLI images showing the tumor burdens at the indicated time points. **C** Tumor burden measured by total flux (top). Survival was analyzed by Kaplan–Meier analysis (middle). Proportion of CAR-T cells among peripheral mononuclear cells on days 7, 12, 19 and 24 (bottom) (mean ± SEM). **D** Cytokines from the sera of blood samples collected 19 days after CAR-T-cell infusion (mean  ±  SEM; two-way ANOVA). All data represent the fold change in the value of the test group compared with the control group, and colors indicate fold changes. **E** Schematic showing the design of the competitive model. CD19-BBζ and CD19-BBζ + CD2 CAR-T cells were labeled with different fluorescent dyes (blue: CellTrace™ Violet dyes; red: CellTrace™ Far Red dyes). **F**, **G** Proportion of different CAR-T cells relative to the total CAR-T-cell population (*n* =  6; mean  ±  SD; two-way ANOVA). ****p  <  0.0001

Next, we designed a multiple-challenge model to better estimate the effect of CD2 overexpression on CAR-T cells. In this model, 5 × 10^5^ Nalm6-L cells were inoculated five days before CAR-T-cell treatment. Following BLI for equalization, 2 × 10^6^ purified CAR-T cells (cultured for 12 days) were infused (Fig. 5A). Within seven days postinfusion, the tumors were nearly eradicated in both treatment groups, with a lower detectable burden in the CD19-BBζ + CD2 CAR-T-cell group. Further challenge with Nalm6-L cells on days seven and twelve resulted in rapid tumor expansion in the CD19-BBζ CAR-T-cell treatment group, while CD19-BBζ + CD2 CAR-T cells continued to effectively restrict tumor growth and prominently improved survival rates (Fig. 5B, C). On day twelve, cytokine levels in the peripheral blood were assessed, revealing elevated cytotoxic protein levels in the CD19-BBζ + CD2 CAR-T-cell group compared with those in the CD19-BBζ CAR-T-cell group (Fig. 5D). Notably, little difference in CAR-T-cell counts between the two treatment groups was observed, which may be attributed to the substantial difference in tumor burden (Fig. 5C). To assess potential off-target toxicity, we performed immunohistochemical staining on organs collected from the mice. The results revealed little abnormal infiltration of CAR-T cells in either conventional CAR-T- or CD2-CAR-T-treated mice (Fig. S8E). Additionally, we cocultured CAR-T cells with K562 cells in vitro and found that overexpressing CD2 did not enhance the cytotoxicity of CAR-T cells toward cells lacking the target antigen (Fig. S8F).

To directly compare the in vivo expansion potential, we differentiated CD19-BBζ and CD19-BBζ + CD2 CAR-T cells with distinct fluorescent dyes, mixed them at a 1:1 ratio (Fig. 5E), and administered a total of 1 × 10^6^ CAR-T cells into Nalm6-L-bearing mice. On days three and seven after CAR-T-cell infusion, we analyzed the ratio of the two CAR-T-cell populations in the peripheral blood and revealed that CD19-BBζ + CD2 CAR-T cells presented a significantly greater proliferative advantage in this competitive model (Fig. 5F). In another solid tumor model, competitive experiments further confirmed that EGFR-BBζ + CD2 CAR-T cells presented greater expansion potential than did EGFR-BBζ CAR-T cells, as evidenced by a greater proportion of CAR-T cells in the peripheral blood and an increased number of CAR-T cells infiltrating the tumor tissue (Fig. 5G).

These results demonstrated that elevated CD2 expression enhanced the antitumor activity and proliferation of CAR-T cells in vivo.

### CD2 overexpression enhances the antigen sensitivity of CAR-T cells

**Fig. 6:**
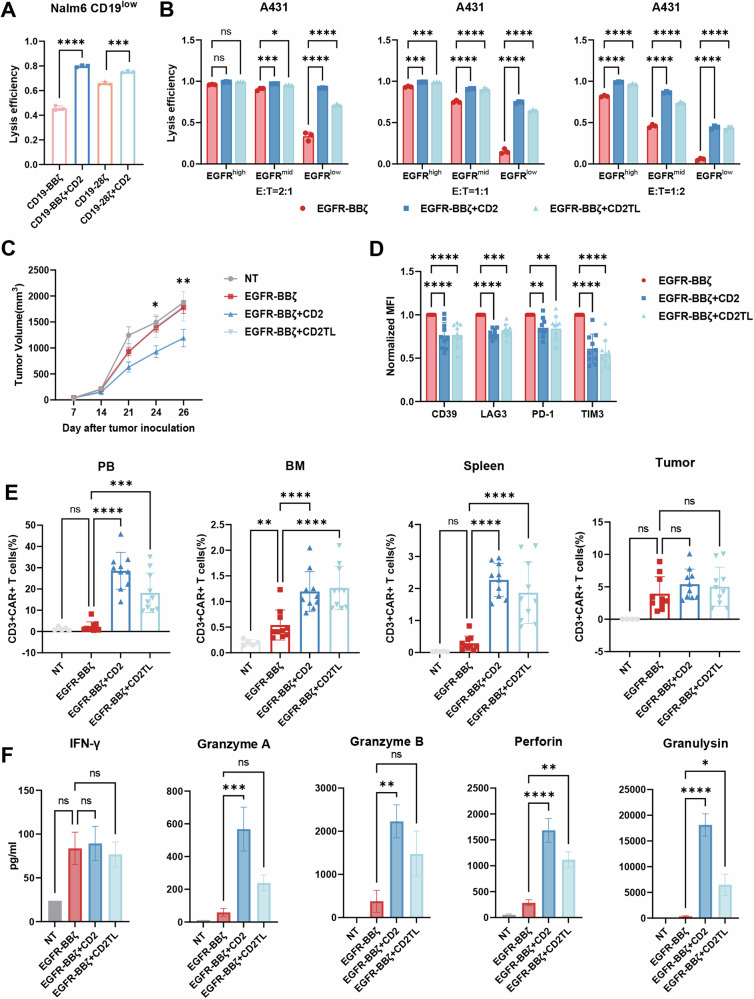
CD2-CAR-T cells demonstrated increased antigen sensitivity. **A** Lysis efficiency of different CAR-T cells against Nalm6 cells with low CD19 expression at an *E*:*T* ratio of 1:10 for 24 h (*n* =  3; mean  ±  SD; two-way ANOVA). **B** Lysis efficiency of different CAR-T cells against A431 cells with varying EGFR levels at the indicated *E*:*T* ratios for 24 h (*n* = 3; mean  ±  SD). **C** Tumor growth monitored in NPG mice inoculated with EGFR-low A431 cells (*n* = 10; mean  ±  SEM; two-way ANOVA). **D** Mean fluorescence intensity (normalized to that of the control) of CD39, PD-1, TIM3 and LAG3 on CAR-T cells (*n*  =  10; mean  ±  SEM; two-way ANOVA). **E** The proportions of CAR-T cells within the peripheral mononuclear cell (PB), bone marrow (BM), spleen and tumor on day 26 were determined via flow cytometry (*n* = 10; mean  ±  SEM; two-way ANOVA). **F** Cytokines from the sera of blood samples on day 26 (*n* =  10; mean  ±  SEM; ordinary one-way ANOVA test). *p  <  0.05, **p  <  0.01, ***p < 0.001, ****p < 0.0001

Drawing from the literature highlighting the correlation between IS quality and antigen sensitivity, we aimed to verify whether CD2 CAR-T cells exhibit increased antigen sensitivity. We initially generated CD19-low Nalm6 and Raji-luc cell lines via the transduction of CD19-null Nalm6 and Raji-luc cells with a lentivirus encoding CD19, followed by CD19 staining and sorting (Fig. S9A). Cytotoxicity assays demonstrated that overexpressing CD2 increased the ability of different CAR-T cells, which employ either 4-1BB or CD28 as the costimulatory domain, to eliminate CD19-low-expressing tumor cells (Fig. 6A). Western blot analysis also demonstrated that under stimulation with low antigen density, overexpressing CD2 significantly enhanced the ability of the CAR molecule to trigger downstream activation signals (Fig. S9B). To further confirm whether CD2-CAR-T cells prevent or delay tumor cell escape triggered by low antigen expression, we established a mouse model by mixing Raji-luc-WT and Raji-luc-CD19-low cells (Fig. S9C) at a 1:1 ratio. As shown in Fig. S9D–G, compared with CAR-T-19 cells, CD19-BBζ + CD2 CAR-T cells presented enhanced tumor suppression efficacy and sustained cellular persistence. Flow cytometric analysis at the experimental endpoint revealed that, compared with conventional CAR-T cells, CD2-overexpressing CAR-T cells accounted for a greater proportion of the CD62L and CCR7 dual-positive population (Fig. S9H). Intriguingly, evaluation of T-cell exhaustion markers revealed divergent trends: CD39 and LAG-3 expression was potentially reduced in CD2-modified cells, whereas TIM-3 and PD-1 expression tended to increase (Fig. S9I). As evidenced by the presented data, there was considerable variability between samples, which made it challenging to achieve statistical significance. A larger sample size may be needed to draw more definitive conclusions.

We further assessed the effect of CD2 overexpression on antigen sensitivity in three A431 cell lines with distinct EGFR expression levels. Cytotoxicity tests confirmed that EGFR-BBζ + CD2 CAR-T cells outperformed EGFR-BBζ CAR-T cells against A431 cells with varying EGFR levels. Notably, the cytotoxicity of conventional CAR-T cells to tumor cells significantly decreased as the antigen density decreased, whereas CD2-CAR-T cells effectively maintained decent sensitivity to low-density antigens (Fig. 6B). Interestingly, overexpression of the extracellular domain of CD2 also significantly increased the degree of cytotoxicity, albeit not reaching the levels observed with full-length CD2. In an additional model mimicking tumor heterogeneity, we mixed the three A431 cell lines at a 1:1:1 ratio (Fig. S10A), which was subjected to 24 h of coculture with CAR-T cells. By calculating the remaining tumor cells, we found that EGFR-BBζ + CD2 CAR-T cells exhibited increased overall cytotoxic efficacy against the entire cell population in this mixed model (Fig. S10B). Notably, conventional CAR-T cells preferentially attacked tumor cells with high and medium EGFR, whereas EGFR-BBζ + CD2 CAR-T cells exhibited a markedly reduced bias (Fig. S10C). No significant difference in the proliferation rate among the three A431 cell lines was observed (Fig. S10D). Quantitative analysis of EGFR and CD19 expression across these cell lines, along with CD58 expression, was also performed (Fig. S10E).

We established a low-antigen-density solid tumor model to evaluate the in vivo antitumor efficacy of EGFR-BBζ + CD2 CAR-T cells. As depicted in Fig. 6C, EGFR-BBζ CAR-T-cell therapy resulted in minimal tumor growth control, while tumor growth in the EGFR-BBζ + CD2 treatment group was markedly suppressed. In this in vivo experiment, CAR-T cells overexpressing the extracellular domain of CD2 did not reproduce the enhanced effect observed in vitro. However, the overexpression of the extracellular domain of CD2 still demonstrated some potential for improving CAR-T-cell performance. For example, similar to EGFR-BBζ + CD2 CAR-T cells, compared with EGFR-BBζ CAR-T cells, these cells both presented lower levels of exhaustion-associated markers on day 19 postinfusion, maintained a greater number of CAR-T cells (not observed in tumor tissue), and secreted greater amounts of cytotoxic granules (Fig. 6D–F).

These results underscore the potential of CD2 overexpression to increase the antigen sensitivity and antitumor efficacy of CAR-T cells, with implications for improving CAR-T-cell therapies against tumors with low antigen density.

## Discussion

CAR-T-cell therapy has revolutionized the treatment of hematological malignancies, with several therapeutic products securing global market approval for B-cell-derived lymphomas, leukemias, and multiple myelomas. Despite this remarkable progress, the potency of CAR-T-cell therapy is often compromised by the suboptimal functionality of T cells, which is characterized by diminished antitumor capabilities and a lack of durability. The next generation of CAR-T cells incorporates a variety of modifications aimed at bolstering their enduring antitumor capabilities. These enhancements include the incorporation of additional costimulatory domains, the optimization of CAR architecture, the expression of cytokines, and the knockout of inhibitory molecules, among others. However, the factors contributing to CAR-T-cell therapy failure are highly variable in real-world settings, and these strategies face numerous challenges before they can be deemed successful.

The immunological synapse, a specialized cell interface between T cells and target cells, is essential for effective T-cell-mediated antitumor responses. It orchestrates a chain of subsequent events, such as cytoskeletal reorganization in T cells, directed release of cytotoxic granules, and activation of downstream signaling pathways that are instrumental in modulating T-cell expansion, metabolic activity, and lineage commitment. For example, a high-quality immune synapse can initiate sufficient downstream activation signals, such as NFκB and AP-1, which are essential for promoting T-cell expansion and survival and for maintaining T-cell fitness. Therefore, enhancing the antitumor efficacy of CAR-T cells by optimizing immunological synapses is widely regarded as a more reliable strategy that is applicable across various scenarios.

We observed that CD2 expression levels were intimately linked to the quality of immunological synapses formed by CAR-T cells and their antitumor potency. This association was further supported by our database analysis, which revealed a positive correlation between CD2 expression levels and the abundance of tumor-infiltrating lymphocytes, as well as an association between high CD2 expression and a prolonged overall survival period across various tumor types. These findings suggest that CD2 expression is not only a marker of T-cell functionality but also a determinant of therapeutic efficacy.

The dynamic regulation of CD2 expression on T cells prompted investigations into its impact on the quality of CAR-mediated synapses, which in turn affects the functional outcomes and differentiation trajectory of CAR-T cells. Our results indicate that as T cells differentiate toward a terminal state, they gradually lose their ability to upregulate CD2 in response to antigenic stimulation. This loss of CD2 expression could hinder the ability of these cells to form high-quality ISs, thereby affecting their antitumor efficacy. To address the suboptimal CD2 expression on T cells, we explored the strategy of exogenous CD2 supplementation. Our study confirmed that sustaining elevated CD2 levels through ectopic expression could optimize CAR-IS, alleviate T-cell exhaustion, and augment the antitumor potential of CAR-T cells. This finding is significant, as it suggests a novel approach to enhance the efficacy of CAR-T-cell therapies by modulating the CD2‒CD58 axis.

The optimization of the IS may augment the antitumor efficacy of CD2 CAR-T cells through several mechanisms: 1. CD2 expression is intricately associated with the ability of CAR-ISs to initiate downstream signaling cascades. Consequently, CAR-T cells with sustained high CD2 + T cells may exhibit increased proliferative potential, as evidenced by their superior in vivo proliferation in competitive models. 2. In addition to simply intensifying the activation signals, an optimized IS may rebalance the multitude of signals downstream of the CAR molecule, potentially improving T-cell fitness in a manner reminiscent of TCR functions. 3. An optimized IS allows CAR-T cells to function in a more “energy-conserving” manner, characterized by a diminished frequency of contacts with tumor cells and a notable reduction in nonlytic contacts. The potential mechanisms underlying this may involve a well-structured IS that can more precisely direct the delivery of cytotoxic granules or extend the duration of IS engagement to ensure complete T-cell cytotoxicity [24]. This shift is expected to mitigate T-cell dysfunction induced by repeated antigen stimulation and to lower the incidence of antigen-low-density tumor cells stemming from nonlytic contacts. 4. The optimized IS increases the sensitivity of CAR-T cells to tumor cells expressing low-density antigens.

Our study confirms the efficacy of CD2 overexpression in potentiating CAR-T-cell therapy; however, several issues warrant further investigation. Research has focused primarily on in vitro and murine models, which may not fully replicate the complexity of the human immune system. It is essential to translate these findings into clinical trials to assess the safety and efficacy of CD2-overexpressing CAR-T cells in human patients. Monitoring potential adverse events, particularly autoimmune responses that may arise from enhanced T-cell activation, is crucial. While overexpression of CD2 enhances the affinity between CAR-T cells and tumor cells, ensuring that this enhancement does not lead to excessive on-target, off-tumor toxicity, potentially harming healthy tissues expressing the target antigen, is vital. Second, the optimal intensity of CAR activation signals may vary across different stages of T-cell differentiation or under different scenarios. Therefore, further research is needed to determine the optimal level of CD2 expression to achieve the correct balance between activation signals, antitumor efficacy, and T-cell fitness. Furthermore, the precise molecular mechanisms underlying the effects of CD2 overexpression require elucidation. On the basis of our data, the mechanical effects generated by the binding of the extracellular domain of CD2 with CD58, as well as the downstream activation signals triggered by the intracellular domain of CD2, both play significant roles in improving immunological synapse function. A deeper understanding of how CD2 interacts with other components of the IS and influences the collaboration of downstream signaling pathways can inform the design of more effective immunotherapies.

## Materials and methods

### Study design

This study employed a controlled experimental design to assess the impact of ectopic CD2 expression in CAR-T cells on the quality of immunological synapses, T-cell exhaustion, and antitumor effects. The underlying hypothesis was that elevated CD2 expression levels would enhance the functionality and durability of CAR-T cells. In vitro experiments utilized a variety of cell lines and primary T cells derived from different healthy donors, whereas in vivo experiments employed murine models of hematological malignancies and solid tumors. Observations included assessments of cytoskeletal reorganization, activation signals, and tumor cell killing efficiency. The measurement techniques included flow cytometry, Western blotting, bioluminescence imaging, and tumor volume measurements. In vivo studies involved the specification of cell or mouse populations and sources, with mice randomly assigned to different treatment and control groups on the basis of tumor burden. The animals were numbered and allocated into groups via a random numbers table or computer-generated random numbers. Researchers who conducted the experiments and evaluated the results were aware of the group allocation, while the animal caretakers and operators were blinded to the treatment assignments. Sample sizes for both in vitro and in vivo experiments were determined on the basis of existing experience and literature standards, without adjustments during the course of the study. Data collection was predefined to cease upon reaching the maximum tumor burden in in vivo models or when significant differences emerged between treatment groups, and in vitro assays were halted upon achieving evident saturation of cytotoxic effects or when significant differences were observed between different treatment groups. Exclusion criteria were applied to outliers, defined as data points lying more than three standard deviations from the mean, established prior to the study’s commencement. The primary endpoints included assessments of immunological synapse quality, T-cell exhaustion markers, tumor lysis efficiency and in vivo tumor burden. The secondary endpoints included cytokine secretion levels and T-cell proliferation rates. Each experiment was repeated at least three times, with the results confirmed under various conditions to ensure reproducibility.

### Cell lines and culture

The cell lines employed in this study were procured from our laboratory’s cell cryopreservation bank. The primary cell lines utilized include human acute lymphoblastic leukemia cells (Nalm6), human Burkitt lymphoma cells (Raji), human non-small cell lung cancer cells (A549), human T-cell leukemia cells (Jurkat), and human skin squamous carcinoma cells (A431). Nalm6-GL and Raji-GL cells have been genetically engineered to express green fluorescent protein (GFP) and luciferase. A549 CD19-GL cells were modified to express high levels of the human CD19 antigen, in addition to GFP and luciferase. A431 EGFR^high^ cells, A431 EGFR^mid^ cells and A431 EGFR^low cells^, provided by Dr. Xin Lin’s laboratory, were engineered to stably exhibit low, medium, and high antigen densities of EGFR and fluorescent proteins, respectively. These cells were cultured in RPMI 1640 or DMEM (Gibco) supplemented with 10% (v/v) fetal bovine serum (FBS, Gibco) at 37 °C and 5% CO_2_.

### Production of lentivirus

For the generation of lentiviral particles, a transfection cocktail was prepared by combining Lipofectamine 3000 (Invitrogen), 12 μg of the core plasmid, 9.6 μg of the viral packaging plasmid psPAX, and 2.4 μg of pMD2G with Opti-MEM (Gibco) at room temperature for a period of 10–20 min. The detailed sequences of the CAR constructs are provided in the Supplementary Materials (Supplementary Table 2). This mixture was then added to 10 cm tissue culture dishes containing 293 T cells. The cells were incubated for 6 h, after which the transfection medium was replaced with 12 mL of complete growth medium (DMEM supplemented with 10% (v/v) FBS, Gibco). An additional 6 mL of complete medium was added after 24 h. Following a 48-h incubation period, the supernatant containing the lentiviral particles was collected and clarified by centrifugation at 2000 RPM for 10 min to remove cellular debris. The resulting lentiviral supernatant was subsequently aliquoted and stored at −80 °C for long-term preservation.

### Generation of CAR-T cells

Healthy human PBMCs were isolated from multiple healthy donors via Ficoll density gradient centrifugation (Cytiva). The T-cell subset was then stimulated with 2 μg/mL CD3 (BioLegend) and CD28 (BioLegend) monoclonal antibodies. For 48 h of activation, the T cells were cultured in X-VIVO™15 medium (Lonza) supplemented with 5% FBS (Gibco). Postactivation, the T cells were infected with lentivirus in the presence of a lentivirus infection-enhancing reagent. The cells were seeded into culture plates and subjected to centrifugation at 850 × g for 2 h at 32 °C to increase viral uptake. After centrifugation, the supernatant was carefully aspirated, and the cells were further cultured in X-VIVO™15 medium supplemented with 5% FBS (Gibco) and recombinant human IL-2 at a concentration of 300 U/mL (Novoprotein) to support T-cell expansion.

### In vitro killing assay

A designated quantity of tumor cells, specifically, 1 × 10^5^ Nalm6-GL cells, 1 × 10^5^ Raji-GL cells, 5 × 10^4^ A431 cells, and 2 × 10^4^ A549 CD19-GL cells—was inoculated into 96-well plates and subsequently cocultured with CAR-T cells to achieve the desired effector-to-target (*E*:*T*) ratio. The control wells, which served as negative controls, contained only tumor cells without CAR-T cells. Following a predetermined duration of coculture, flow cytometry analysis was performed to determine the number of viable tumor cells. The cytolytic efficiency was then determined via the following formula.

lysis% = (1 − (treatment count/negative control count)) × 100

For multiround killing assays, CAR-T cells and tumor cells were cocultured at predetermined E:T ratios. Following 72 h of coculture, the tumor cells were nearly completely eradicated. Subsequently, viable residual CAR-T cells were isolated via fluorescence-activated cell sorting (FACS) and subjected to subsequent rounds of coculture. After the final round of coculture, the CAR-T cells were maintained in complete medium supplemented with IL-2 for an additional 72 h prior to analysis of surface marker expression.

### Proliferation assay

Six-well plates were inoculated with 1 × 10^6^ CAR-T cells and incubated in X-VIVO™15 medium (Lonza) supplemented with 5% FBS (Gibco) and 300 U/mL recombinant human IL-2 (PeproTech). Cell enumeration was conducted at 48-h intervals over a period of four sequential time points.

### Flow cytometry analysis

Cells were labeled with fluorochrome-conjugated monoclonal antibodies in phosphate-buffered saline (PBS) supplemented with 2% FBS (Gibco). The specific monoclonal antibodies utilized are shown in Supplementary Table 1. Isotype-matched control mAbs were included in all procedures to ensure specificity. The LEGENDplex™ Multi-Analyte Flow Assay Kit Human CD8/NK Panel (13-plex) with V-bottom Plate V02 (BioLegend) was used in accordance with the manufacturer’s experimental protocol to assess cytokine secretion levels. Flow cytometric analysis of the samples was performed via a Beckman CytoFLEX flow cytometer, and subsequent data analysis was conducted via FlowJo software.

### Quantification of antigen utilizing QuantiBrite beads

The BD Quantibrite™ PE assay tube was reconstituted with 0.5 mL of diluent (phosphate-buffered saline containing sodium azide supplemented with 0.5% bovine serum albumin) and subjected to vortex mixing to ensure homogeneity prior to application. Flow cytometric analysis was performed in conjunction with the generation of a standard curve to ascertain the quantitative correlation between antigen concentration and PE fluorescence intensity. The sample cell density was standardized to 2 × 10^5^ cells/mL in PBS, followed by the addition of a mixture consisting of 100 µL of this cell suspension and 4 µL of either anti-CD19 PE-conjugated or anti-EGFR PE-conjugated fluorescent antibody. This mixture was then incubated at 2–8 °C for 30 min in a dark environment to allow for antibody binding. After incubation, the cells were washed twice with 1 mL of 1× PBS solution and centrifuged at 300 × *g* for 5 min. The pellet was resuspended in 200 µL of 1× PBS and subsequently analyzed via a flow cytometer. The antigen quantity on the cells was determined by measuring the specific fluorescence intensities associated with the PE label.

### Monitoring the dynamic interactions between CAR-T cells and tumor cells

CAR-T cells cultured for 10 days were harvested with 1× PBS. A 2× cell suspension was prepared by adding 1 mL of Diluent C (Sigma‒Aldrich) and gently resuspending the cells by pipetting, avoiding vortexing or prolonged exposure to Diluent C. A 2× dye solution was prepared in Diluent C by mixing 4 μL of PKH67 (Sigma‒Aldrich) dye with 1 mL of Diluent C in a centrifuge tube, ensuring thorough mixing. The 2× cell suspension was then combined with the prepared 2× dye solution and mixed continuously by pipetting for 2–3 min, ensuring that the final cell concentration did not exceed 1 × 10^7^ cells/mL. The staining process was terminated by adding 2 mL of 10% FBS and incubating for 1 min to facilitate the binding of excess dye. The CAR-T cells were centrifuged, and the cell pellet was resuspended in complete media. A total of 1 × 10^6^ stained CAR-T cells were plated onto 12-well tissue culture plates in 1 mL of media and incubated overnight. Raji cells, which were stained with the CellTrace Far Red Cell Proliferation Kit (Thermo Fisher Scientific), were then added and cocultured with the stained CAR-T cells at a target ratio of 1:10 for 6 h. During this incubation period, samples of the cell suspension were collected every 2 h for flow cytometric analysis.

### Cell binding avidity assay

Nalm6 cells were cultured to achieve 70–80% confluence. The cells were subsequently harvested, washed with PBS, and centrifuged at 1500 RPM for 5 min to prepare a single-cell suspension with a concentration of 1.8 × 10^8^ cells/mL in RPMI 1640 medium. The assay chip was precoated with 0.002% poly-L-lysine solution (Sigma‒Aldrich) and then loaded with 20 µL of the cell suspension into the z-Movi® Cell Avidity Analyzer’s cell reservoir. The chip was incubated for 2 h at 37 °C to allow for cell attachment. Jurkat CAR-T cells were labeled with the CellTrace Far Red Cell Proliferation Kit (Thermo Fisher Scientific) prior to the experiments. A suspension containing 5 × 10^6^ Jurkat CAR-T cells was introduced into the chip and incubated for 5 min to facilitate cell binding. Following incubation, the Jurkat cells were dissociated from the chip via the z-Movi system, which employs acoustic and fluidic shear forces. Data analysis was conducted via Ocean software version 1.2.8, whereas statistical evaluation was performed via Prism GraphPad software.

### Western blot analysis

For immunoblotting, 1 × 10^6^ Jurkat-CAR-T cells were cocultured with 1 × 10^6^ target cells. The cell mixture was lysed on ice for 15–25 min in 200 µL of RIPA buffer (Thermo Fisher Scientific) with freshly added protease (Thermo Fisher Scientific) and phosphatase inhibitor cocktail (MedChemExpress), followed by centrifugation at 12,000 × *g* for 30 min at 4 °C to clear the lysate. The samples were then denatured in SDS‒PAGE sample buffer and incubated at 95 °C for 5 min prior to being resolved by SDS‒polyacrylamide gel electrophoresis (PAGE) (GenScript) with equal volumes of each sample. Proteins were subsequently transferred onto polyvinylidene difluoride (PVDF) membranes (Immobilon®) according to standard protocols and blocked with 5% (w/v) BSA (Solarbio®) in Tris-buffered saline containing 0.1% Tween-20 (TBST) for 1 h at room temperature. The primary antibody was diluted appropriately in TBST and incubated with the membrane overnight at 4 °C. The membranes were then washed 10 times with TBST, followed by incubation with a 1/5000 dilution of horseradish peroxidase (HRP)-conjugated secondary antibody for 1 h at room temperature. After secondary antibody incubation, the membranes were washed 10 times with TBST on a shaker. Finally, the blots were developed via chemiluminescence detection reagents according to the manufacturer’s instructions, and the signals were captured via chemiluminescence imaging systems (Servicebio SCG-W3000). Antibody details are provided in Supplementary Table 1. The images were processed with ImageJ 1.51j8 for uniform brightness/contrast adjustment.

### Competitive adhesion assay

A549 CD19-GL cells were seeded on coverslips in 24-well plates at a density of 5 × 10^5^ cells per well. EGFR-BBζ CAR-T cells and EGFR-BBζ + CD2 CAR-T cells were individually labeled with CellTrace™ Far Red dyes (Thermo Fisher Scientific) or CellTrace™ Violet dyes (Thermo Fisher Scientific) and then mixed in equal proportions. The resulting cell suspension was adjusted to a concentration of 1 × 10^6^ cells/mL, and 1 mL of this suspension was added to each well. Following coculture for 30 min in a 5% CO_2_ incubator at 37 °C, the coverslips were carefully removed, and the cells were washed three times with PBS to remove nonadherent cells. The coverslips were subsequently fixed in a solution of 4% paraformaldehyde at room temperature for 15 min, followed by another round of washing with PBS before being sealed with 95% glycerol. Finally, the cells were examined under a fluorescence microscope, and the number of cells within ten random fields at ×200 magnification was quantified via GraphPad Prism software for statistical analysis.

### Immunofluorescence confocal microscopy

After tumor cells were labeled with CellTrace™ Violet dyes (Thermo Fisher Scientific), a total of 1 × 10^6^ cells were gently seeded onto a 15 mm glass bottom cell culture dish (NEST) precoated with 0.1% poly-L-lysine (Sigma‒Aldrich). Once a confluent cell layer was established, an equal number of Jurkat-CAR-T cells were introduced, and the coculture was incubated for 30 min in a 5% CO_2_ incubator at 37 °C. The cells were then fixed with 4% paraformaldehyde for 10 min, followed by permeabilization with a PBS solution containing 0.5% Triton X-100 for 5 min at room temperature. After three washes with PBS, the cells were incubated with 200 µL of prepared TRITC-labeled phalloidin working solution (Yeasen) in the dark at room temperature for 30 min. Following further washes with PBS, fluorescence was visualized under a confocal microscope via a TRITC excitation/emission filter (Ex/Em = 545/570 nm) and a Violet excitation/emission filter (Ex/Em = 405/450 nm). To assess the formation of immune synapses, more than 30 conjugates, representing direct cell‒cell contact between effector cells (Jurkat CAR-T cells or CAR-T cells) and target cells (tumor cells), were randomly selected on each dish. The polarization of immune synapses was quantified by calculating the ratio of the fluorescence intensity of F-actin at the conjugate interface to that of the unconjugated membrane. Nalm6 cells stably expressing GFP and Jurkat-CAR-T cells labeled with CellTrace™ Violet (Thermo Fisher Scientific) were cocultured at a 1:1 ratio for 30 min at 37 °C. The cells were fixed for 15 min at room temperature and stained with an anti-CD45-APC antibody (BioLegend) for 30 min on ice. After washing, the cells were imaged via a Leica TCS SP8 confocal microscope equipped with 405 nm (CellTrace™ Violet), 488 nm (GFP), and 640 nm (APC) lasers. Statistical analysis was conducted via GraphPad Prism software [42].

### RNA sequencing analysis

For RNA sequencing (RNA-seq), CAR-T cells cocultured with Raji cells at a target ratio of 1:5 were sorted via an SH800S Cell Sorter (Sony Biotechnology). The RNA-seq analysis was conducted on the Illumina NovaSeq platform. Quality assessment of the sequencing reads was performed via FastQC, followed by alignment of the single-ended reads to the human reference genome (hg38) via HISAT2 (version 2.2.1). The R statistical software environment was utilized for subsequent statistical analyses. Principal component (PC) analysis was executed with the gmodels R package. The statistical significance of the DEGs was determined via the DESeq2 R package (version 1.32.0). Processed RNA-seq datasets were retrieved from the GEO database (accession number GSE9650) to generate gene set enrichment analysis (GSEA) plots. The raw RNA sequencing data generated in this study have been deposited in the NCBI Gene Expression Omnibus (GEO) database and are accessible under the accession number GSE291458.

### Study approval

All the animal experiments were approved by the Institutional Animal Care and Use Committee (IACUC) of Chinese PLA General Hospital (PLAGH), and all the procedures were performed in accordance with the guidelines of the IACUC of PLAGH.

### Statistical analysis

Data analysis and visualization were performed via GraphPad Prism 9 software. Statistical tests were performed via unpaired Student’s t tests, one-way ANOVA, or two-way repeated-measures ANOVA to compare significant differences. Survival analysis was performed via the log-rank (Mantel‒Cox) test. The graphs represent individual values ± SEMs or ± SDs for in vitro and in vivo experiments. The statistical tests employed for calculating the P values are described in the corresponding figure legends. Significance was set at p < 0.05, with p values denoted by asterisks as follows: not significant (ns), *p < 0.05, **p < 0.01, ***p < 0.001, and ****p < 0.0001.

## Supplementary information

Supplementary material-marked-up

Supplementary Table 1

Supplementary Table 2

supplementary_WB_raw

Original uncropped Western blot membranes corresponding to Figure S9B, Figure 2F and Figure 4G
