# Supplementary material for: CD2 augmentation enhances CAR-T-cell efficacy via immunological synapse remodeling and T-cell exhaustion mitigation
Source: Cell Mol Immunol. 2025 Jul 4;22(8):935–48. doi: 10.1038/s41423-025-01314-6 (PMC12311108; doi:10.1038/s41423-025-01314-6)
Supplement: Supplementary file 1 — Supplementary material-marked-up [file 41423_2025_1314_MOESM1_ESM.docx]

CD2 Augmentation Enhances CAR-T Cell Efficacy via Immunological Synapse Remodeling and T Cell Exhaustion Mitigation

Qi Zhu, Jiajia Li, Nan Liu, Lu Han, Zhiqiang Wu, Yao Wang, Xin Lin*, Jianshu Wei*, Weidong Han*


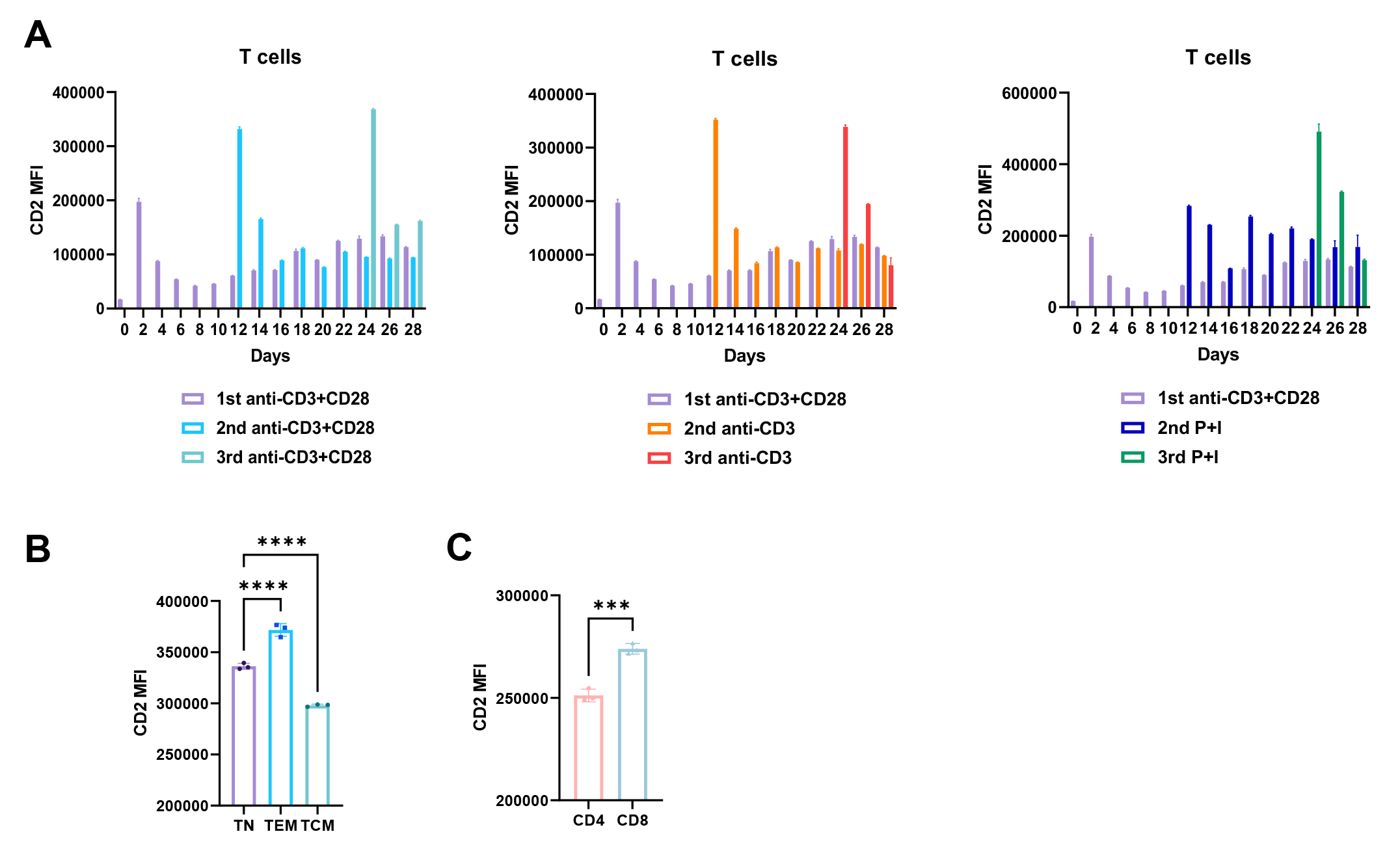


Figure S1. The dynamical expression pattern of CD2 on T cells. (A) The dynamics of CD2 expression on T cells under repetitive stimulation with 2μg/ml CD3 and CD28 monoclonal antibodies, 2μg/ml CD3 monoclonal antibodies, or PMA (100 ng/ml) plus ionomycin (1 μg/ml) (P + I) (n = 3; mean ± SD). (B) The expression level of CD2 in various T cell subsets (TN: naive T cells (CD62L^+^ CD45RA^+^), TEM: effector memory T cells (CD62L^–^ CD45RA^–^), TCM: central memory T cells (CD62L^+^ CD45RA^–^) on the 10th day after initial activation by CD3 + CD28 antibodies (n = 3; mean ± SD; one-way ANOVA test). (C) The expression level of CD2 in CD4^+^ T cells and CD8^+^ T cells on the 10th day after initial activation by CD3 + CD28 antibodies (n = 3; mean ± SD; unpaired t-test). ****p* < 0.001, *****p* < 0.0001.


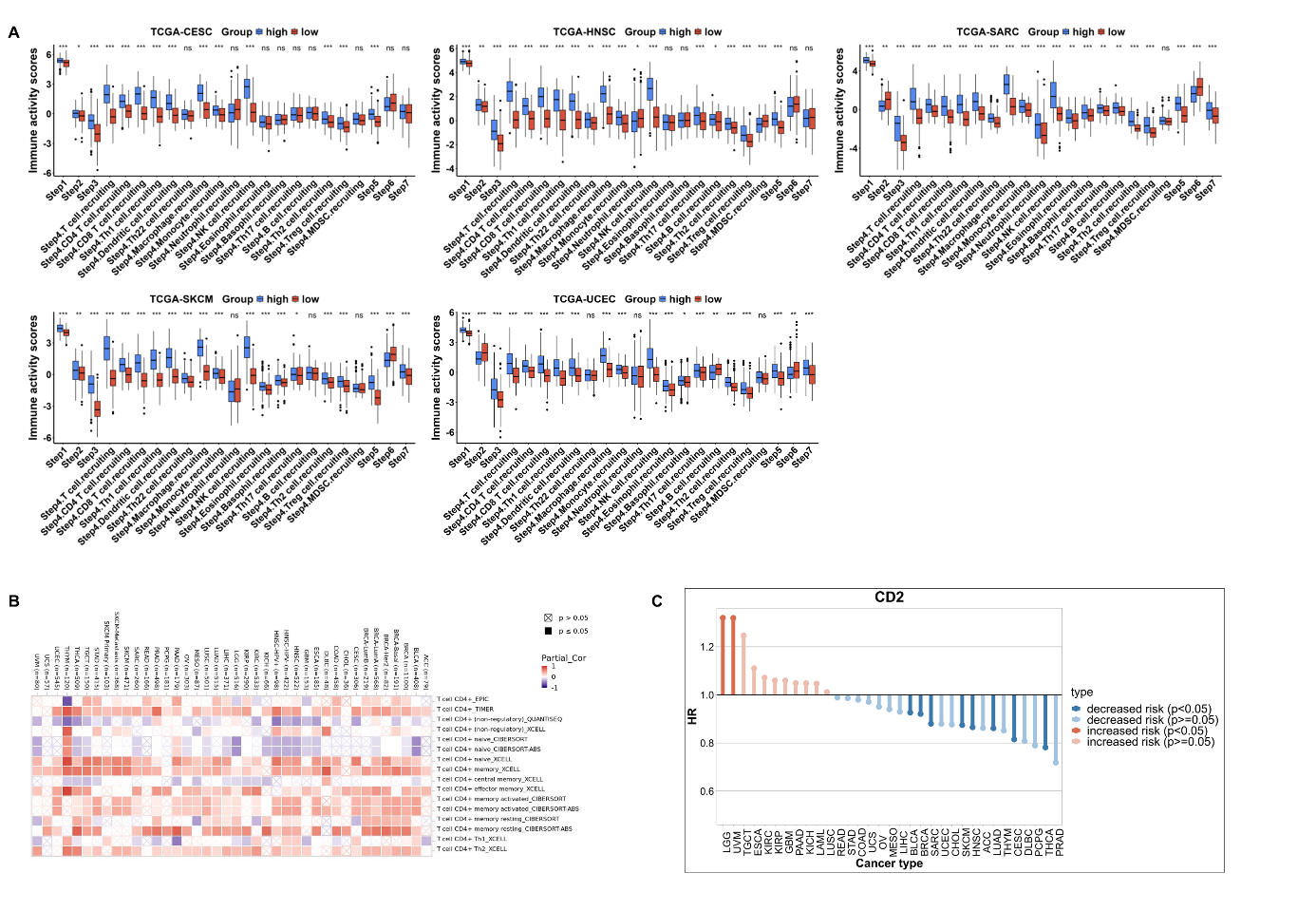


Figure S2. CD2 expression correlates with cancer-immunity cycle activity, tumor-infiltrating lymphocyte abundance, and clinical prognosis across multiple tumor types. (A) CD2 expression and its association with cancer-immunity cycle activity across multiple tumor types. This figure explores the relationship between CD2 expression and cancer-immunity cycle activity using the Tumor Immune Phenotype (TIP) database. The analysis was performed across five tumor types from The Cancer Genome Atlas (TCGA): CESC, HNSC, SARC, SKCM, UCEC. The Mann-Whitney U test was used to assess differences. (B) Correlations of CD2 expression with CD4^+^ T cell immune infiltration in cancers (data from TIMER2). The heatmap presents the adjusted Spearman rank correlation coefficients across various cancer types. (C) The graph shows the expression levels of CD2 and the survival risk, indicating that high expression of CD2 is positively correlated with reduced mortality risk (data from TISIDB). HR: Hazard ratio. The color represents significance; red and blue indicate significant results, while light red and light blue indicate insignificant results. **p* < 0.05, ***p* < 0.01, ****p* < 0.001.


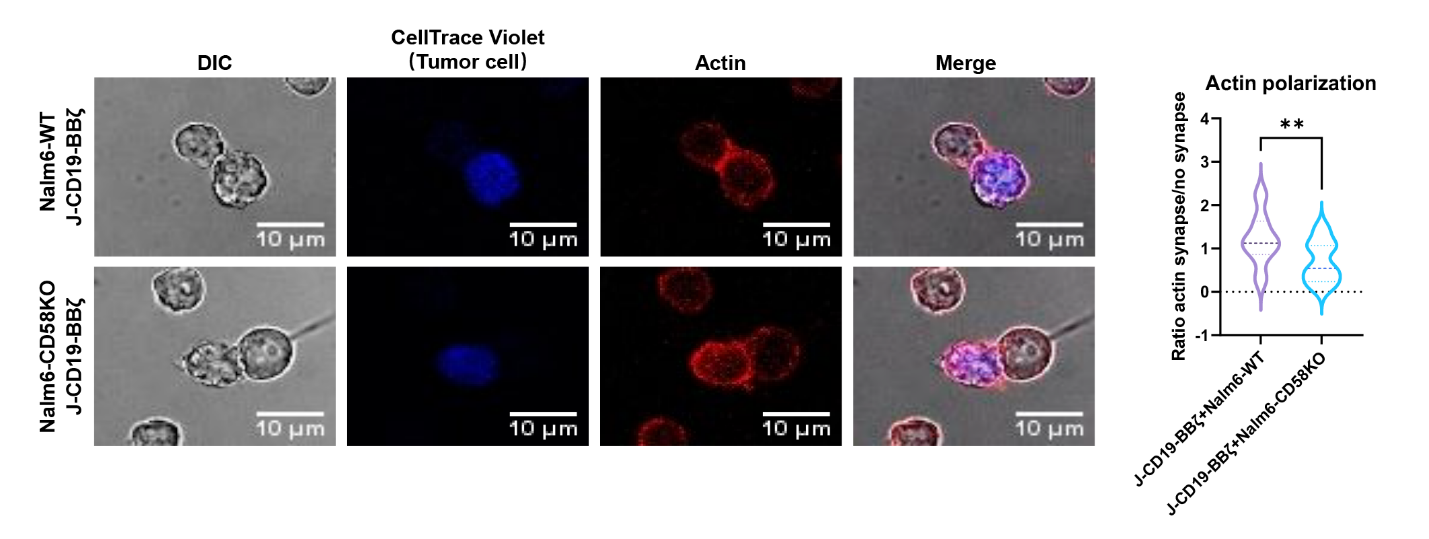


Figure S3. The absence of CD58 on tumor cells impaired CAR-IS. Representative images obtained for the analysis of actin polarization at the IS and quantification of actin polarization by measuring the ratio of F-actin inside and outside the synapse (n = 30; mean ± SEM; Scale bar = 10 μm; unpaired t-test). ** *p* < 0.01.


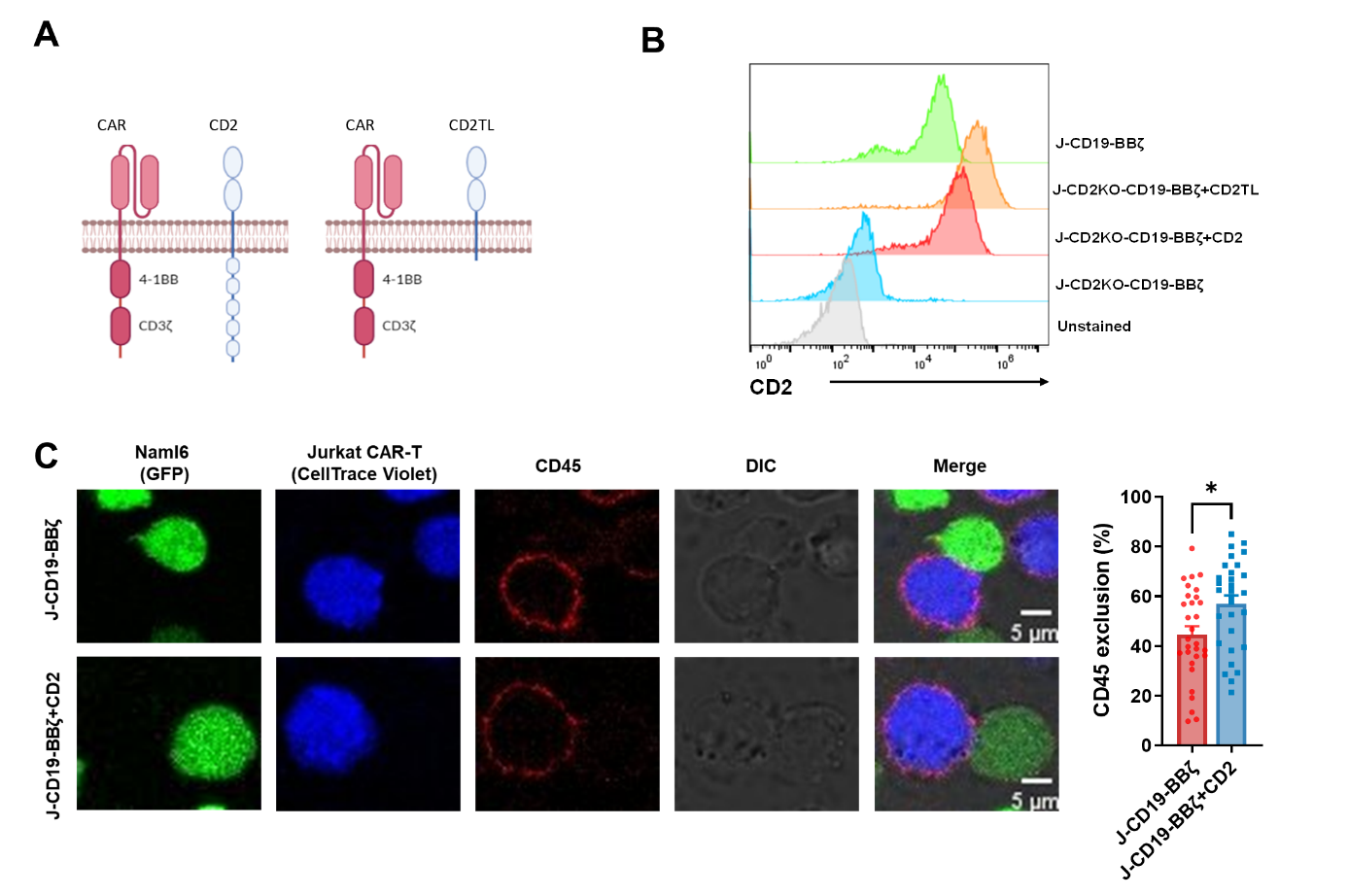


Figure S4. CD2 overexpression improved the exclusion of CD45 from IS area in Jurkat CAR-T cells. (A) Schematic representations of co-expression of CAR structures and full-length CD2 or the CD2 extracellular domain. (B) Representative FACS histograms showing CD2 expression levels in Jurkat CAR T cells (J-CD19-BBζ), CD2-null Jurkat CAR T cells (J-CD2KO-CD19-BBζ), CD2-null Jurkat CAR T cells rescued with the CD2 extracellular domain (J-CD2KO-CD19-BBζ+CD2TL), and CD2-null Jurkat CAR T cells rescued with full-length CD2 (J-CD2KO-CD19-BBζ+CD2). (C) Representative confocal microscopy images showing CD45 exclusion at CAR-T cell immune synapses (left). Quantification of CD45 exclusion percentage (right). Jurkat CAR-T cells and Nalm6 cells (E: T = 1:1) were cocultured for 30 min and stained with an anti-CD45 antibody conjugated with APC. The exclusion percentage = (1 − I_CD45 in car zone_/I_CD45out car zone_) × 100%.CD45 (n = 30; mean ± SEM; Scale bar = 5 μm; unpaired t-test). **p* < 0.05,


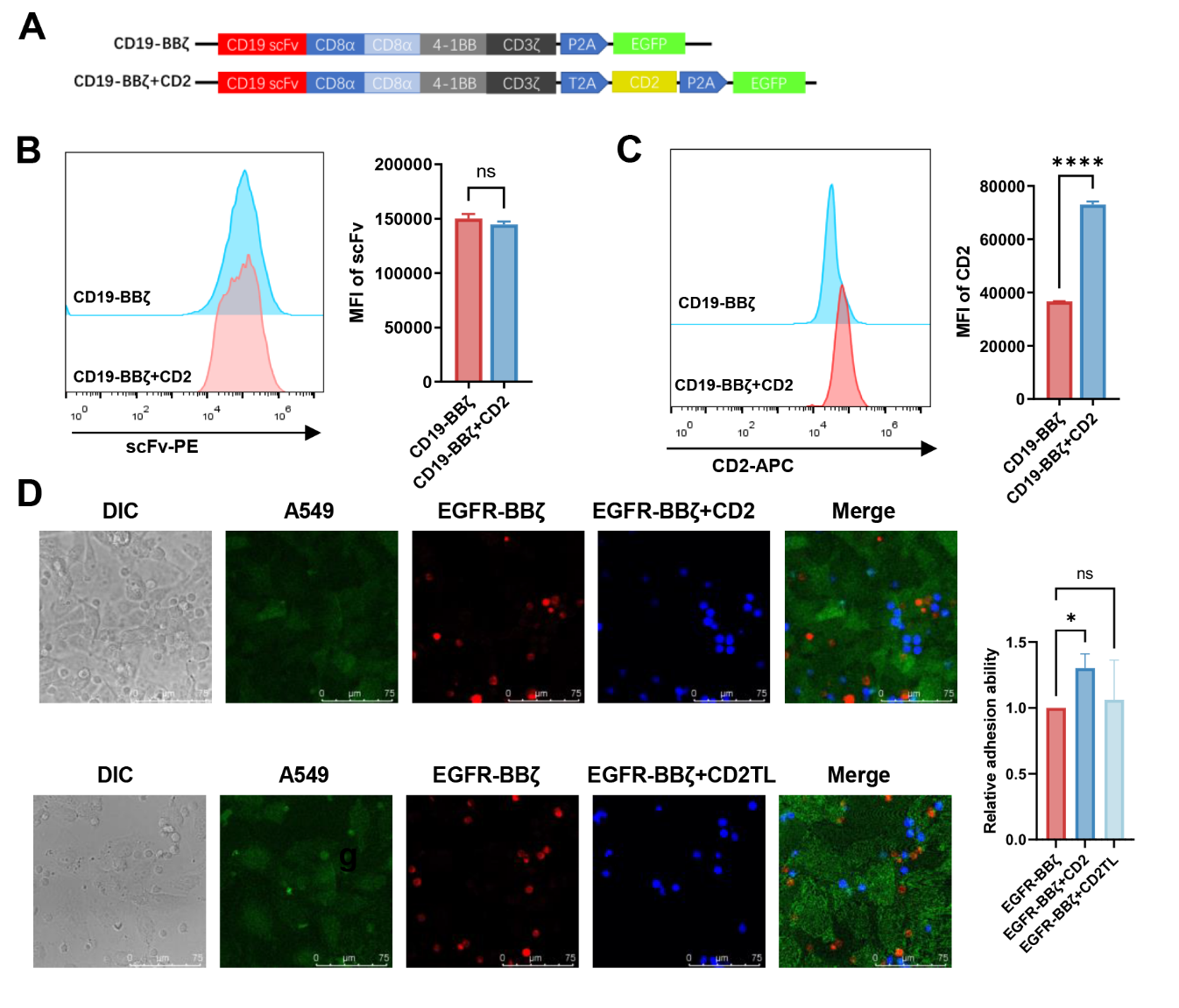


Figure S5. Ectopic expression of CD2 and its impact on binding ability of CAR-T cells. (A) Schematics of CD19-BBζ and CD19-BBζ+CD2 CAR. (B) Representative FACS histogram of CAR expression in CD19-BBζ and CD19-BBζ+CD2 CAR T cells (left). Staining of CAR with scFv-specific antibodies. Quantification of mean fluorescence intensity of scFv (right) (n = 3; mean ± SD; unpaired t-test). (C) Representative FACS histogram of CD2 expression in CD19-BBζ and CD19-BBζ+CD2 CAR T cells (left). Quantification of CD2 mean fluorescence intensity (right) (n = 3; mean ± SD; unpaired t-test). (D) Representative images (left) and relative adhesion ability of competitive adhesion assay (right). Relative adherence was calculated as a fold change compared with the EGFR-BBζ group (n = 10; mean ± SEM; Scale bar = 75 μm; one-way ANOVA test). **p* < 0.05, *****p* < 0.0001.


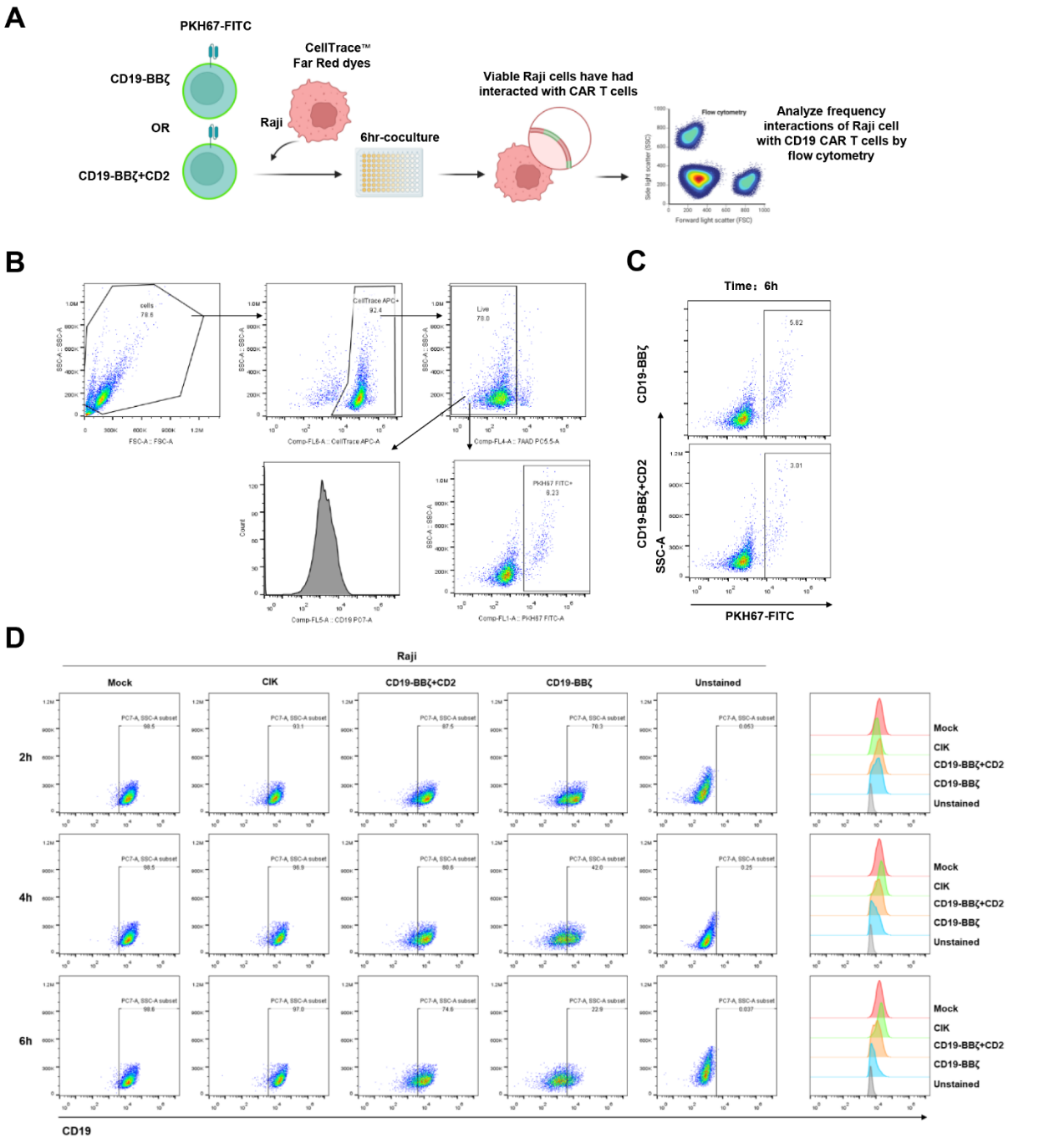


**Figure S6**. The dynamics of interactions between CAR-T cells and tumor cells. **(A)** Experimental design for monitoring the dynamic interaction between CAR T cells and tumor cells. **(B)** Flow cytometry gating strategy. **(C)** Representative FACS plots showing the PHK67 transfer. **(D)** Representative FACS plots showing CD19 expression in viable Raji cells that were co-cultured with CAR T or CIK cells.


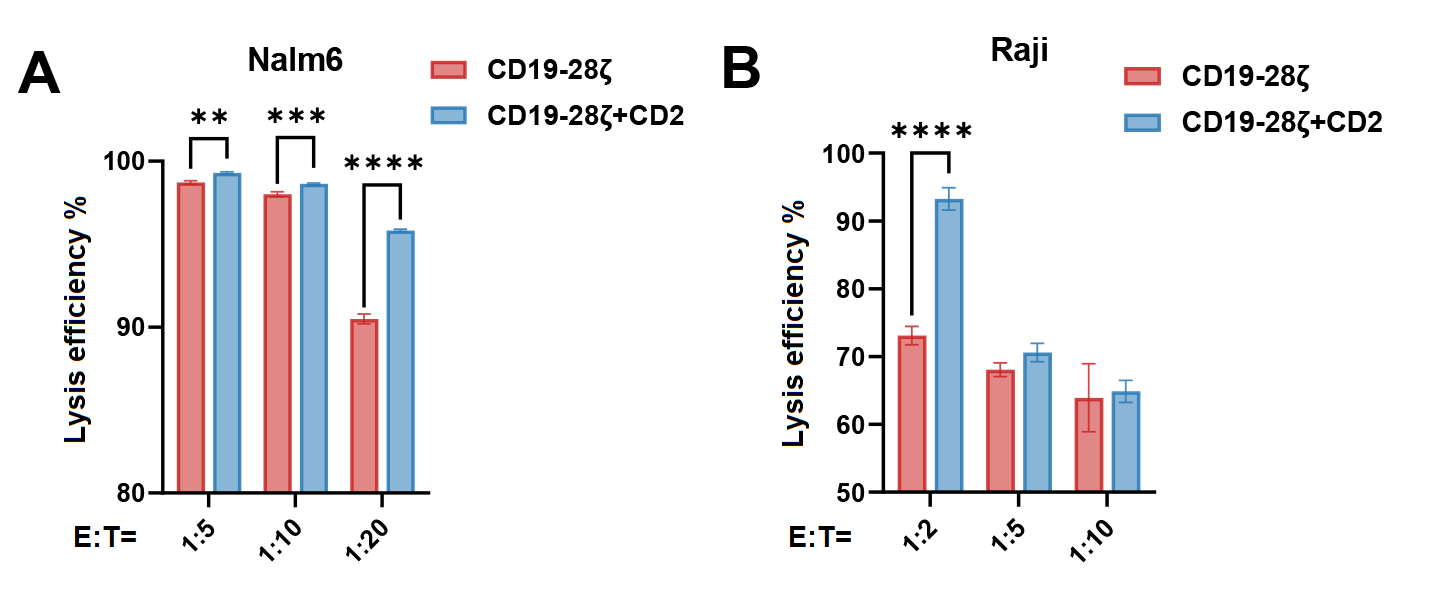


Figure S7. The lysis efficiency of CAR-T cells employing CD28 as costimulatory domain. Cytotoxic analysis of CD19-28ζ and CD19-28ζ+CD2 CAR T cells co-cultured with Nalm6 cells (A) and Raji cells (B) at indicated E:T ratios for 24 hours (n = 3; mean ± SD; two-way ANOVA test). ***p* < 0.01, ****p* < 0.001, *****p* < 0.0001.


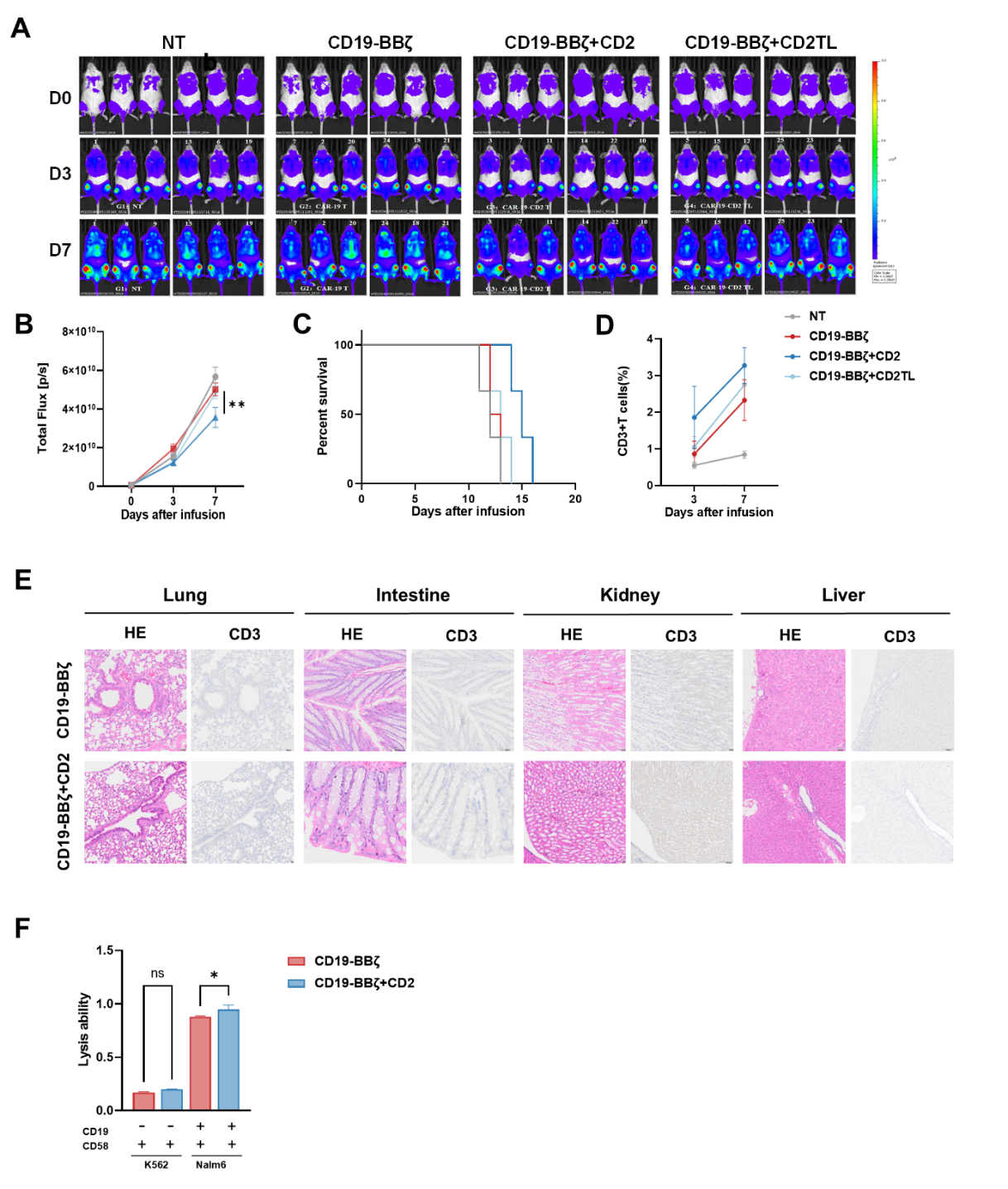


Figure S8. The results of acute leukemia models. (A) The tumor burden presented by BLI images. (B) Tumor burden presented by total flux (mean ± SD; two-way ANOVA test). (C) Survival was analyzed by Kaplan–Meier analysis. (D) The proportion of CAR-T cells in peripheral blood (mean ± SEM). (E) Immunohistochemical analysis of off-target CAR-T cell infiltration in major organs. Representative images of lung, intestine, kidney, and liver sections from NPG mice treated with CD19-BBζ or CD19-BBζ+CD2 CAR-T cells. Tissues were stained with anti-human CD3 antibody (brown) to detect CAR-T cells. (scale bar = 100 μm). (F) Lysis efficiency of CD19-BBζ and CD19-BBζ+CD2 CAR-T cells against K562 cells and Nalm6 cells at an E: T ratios of 1:5 for 24 hours (n = 3; mean ± SD; two-way ANOVA test) **p* < 0.05, ***p* < 0.01.

**
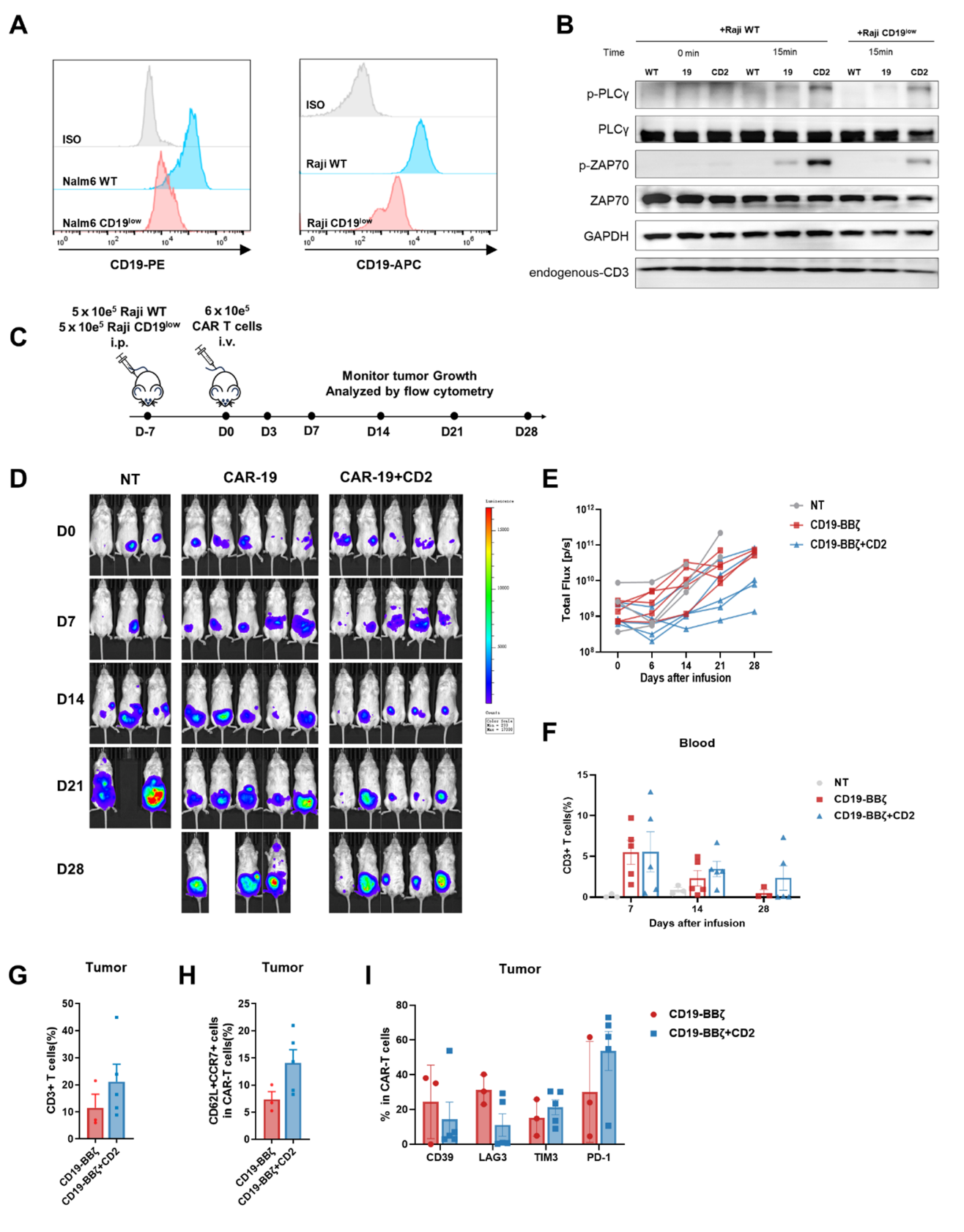
**

**Figure S9.** Overexpression of CD2 enhances CAR-T cell sensitivity to low-density antigens. **(A)** Representative FACS histogram of CD19 expression level in Isotype Control (ISO), Nalm6 WT, Nalm6 CD19^low^, Raji WT, and Raji CD19^low^ cells. **(B)** Proximal signaling events of Jurkat cells (Marked as WT), J-CD19-BBζ (Marked as 19), and J-CD19-BBζ+CD2 (Marked as CD2) CAR-T cells cocultured with Raji WT, or Raji CD19^low^ cells. Image is uniform brightness/contrast adjusted, see supplementary materials for raw data. **(C)** Schematic of the Raji-WT/Raji CD19^low^ mixed tumor model (1:1 ratio). BLI and flow cytometry was performed at the indicated time points. **(D)** BLI images showing tumor burdens at the indicated time points. **(E-F)** Tumor burden and CAR-T cell expansion over time (n = 5; mean ± SEM; two-way ANOVA test). **(G)** Proportion of CAR-T cells within tumors on day 28 (CD19-BBζ group, n = 3; CD19-BBζ+CD2 group, n = 5; mean ± SEM; unpaired t-test). **(H-I)** Memory markers (CD62L, CCR7) and exhaustion markers (CD39, LAG3, TIM3, and PD-1) on CAR-T cells at tumor (CD19-BBζ group, n = 3; CD19-BBζ+CD2 group, n = 5; mean ± SEM; unpaired t-test).


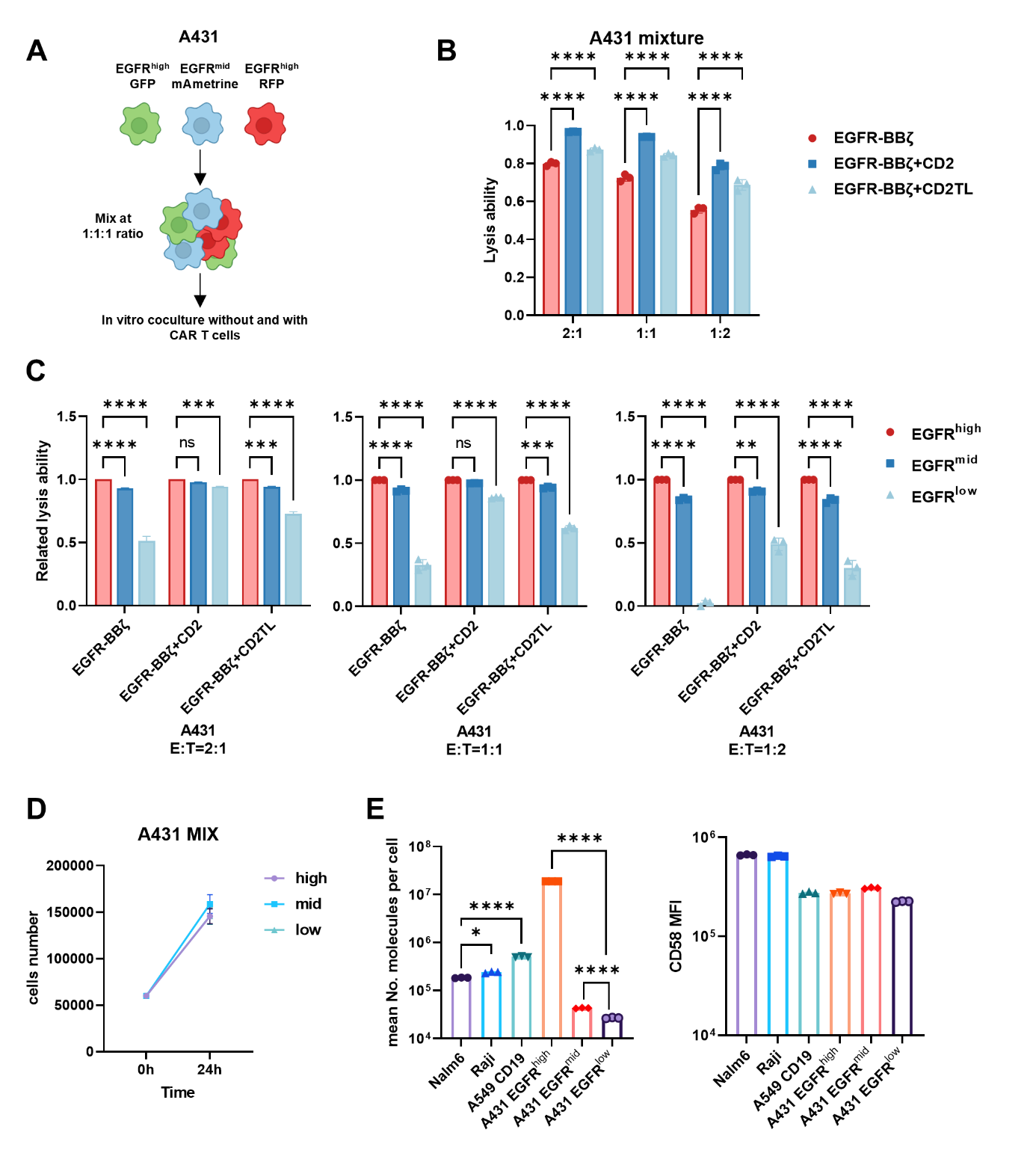


Figure S10. EGFR-BBζ + CD2 CAR-T cells exhibited higher sensitivity to antigen-low tumors. (A) Experimental design of the A431 cells mixture model. (B) Cytotoxic analysis of against the mixture of A431 cells with different EGFR expressions at the indicated E:T ratios for 24 hours (n = 3; mean ± SD; two-way ANOVA test). (C) Statistical analyses of the killing abilities of CAR-T cells against three different A431 cell types, and use the killing efficiency against EGFR-high A431 cells as the reference (normalized to 1) (n = 3; mean ± SD; two-way ANOVA test). (D) Cell growth of different A431 cells in the mixture model (n = 3; mean ± SEM). (E) Quantitative analysis of CD19, EGFR and CD58 across different cell lines. (n = 3; mean ± SD). **p* < 0.05, ***p* < 0.01, ****p* < 0.001, *****p* < 0.0001.
