# Supplementary material for: CD2 augmentation enhances CAR-T-cell efficacy via immunological synapse remodeling and T-cell exhaustion mitigation
Source: Cell Mol Immunol. 2025 Jul 4;22(8):935–48. doi: 10.1038/s41423-025-01314-6 (PMC12311108; doi:10.1038/s41423-025-01314-6)
Supplement: Supplementary file 4 — supplementary_WB_raw [file 41423_2025_1314_MOESM4_ESM.docx]

1. The WB images presented in the manuscript were acquired using a standard imaging system that generated three outputs: brightfield (protein marker), darkfield (chemiluminescent signal), and a composite overlay. To improve visual clarity and ensure accurate interpretation of the results, global brightness adjustments were applied exclusively to the brightfield channel. This adjustment was performed uniformly across the entire image without any selective/local modifications (e.g., cropping, erasing, or region-specific alterations). The original chemiluminescent signal (darkfield channel) and composite image remain unaltered. Red boxes indicate the regions displayed in the main figures.
2. For the main text Figure 2D, the CAR CD3 band (indicated by the red box in this document page 5) was proportionally scaled using ImageJ 1.51j8 without changing the aspect ratio, to match the physical width of the adjacent bands. This operation did not alter the band's grayscale values, relative positions, or background signal.
3. We affirm that these adjustments were strictly limited to optimizing visualization and do not affect the scientific integrity or interpretation of the data.


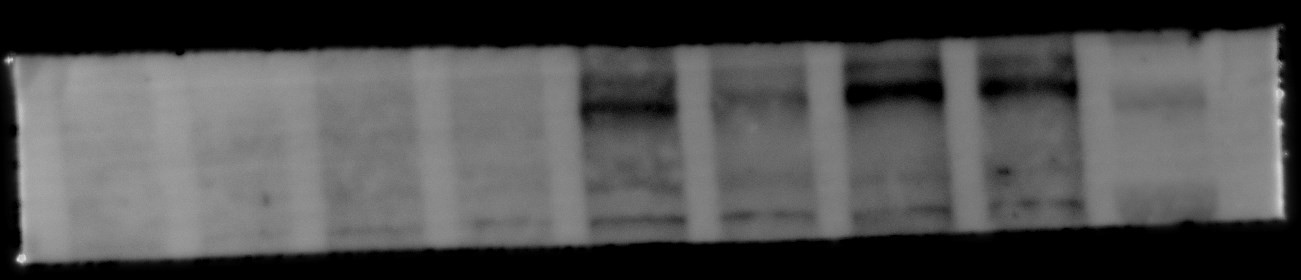

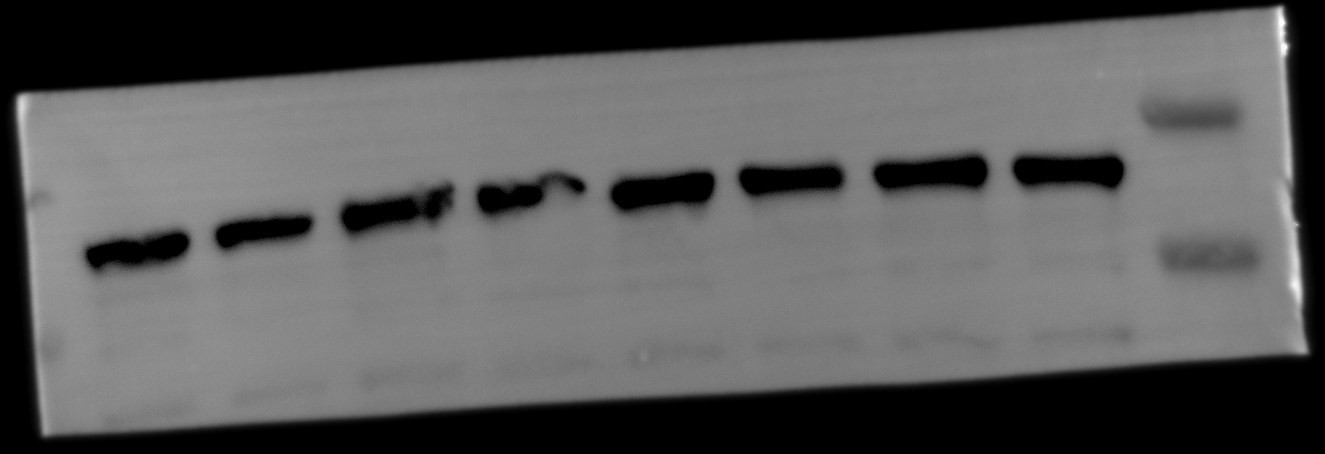


Jurkat CAR T+Nalm6 WT

15 min

0 min

19

KO

CD2

TL

19

KO

CD2

TL

**130kd**

p-PLCγ

**100kd**

**130kd**

PLCγ

**100kd**


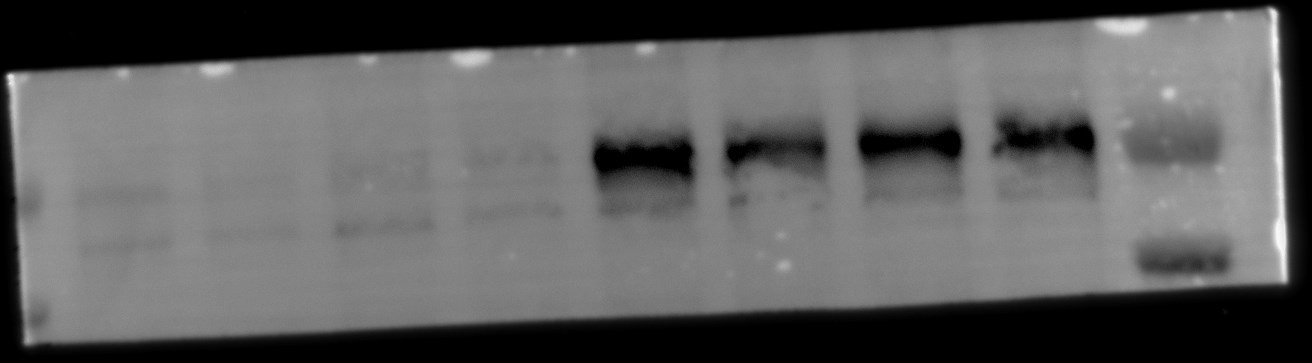

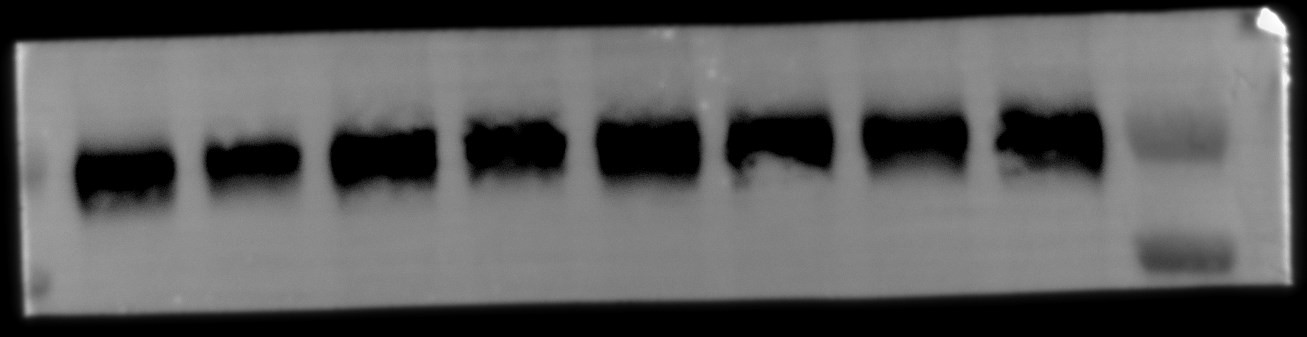


Jurkat CAR T+Nalm6 WT

0 min

15 min

19

KO

CD2

TL

19

KO

CD2

TL

**70kd**

p-ZAP70

**55kd**

**70kd**

ZAP70

**55kd**


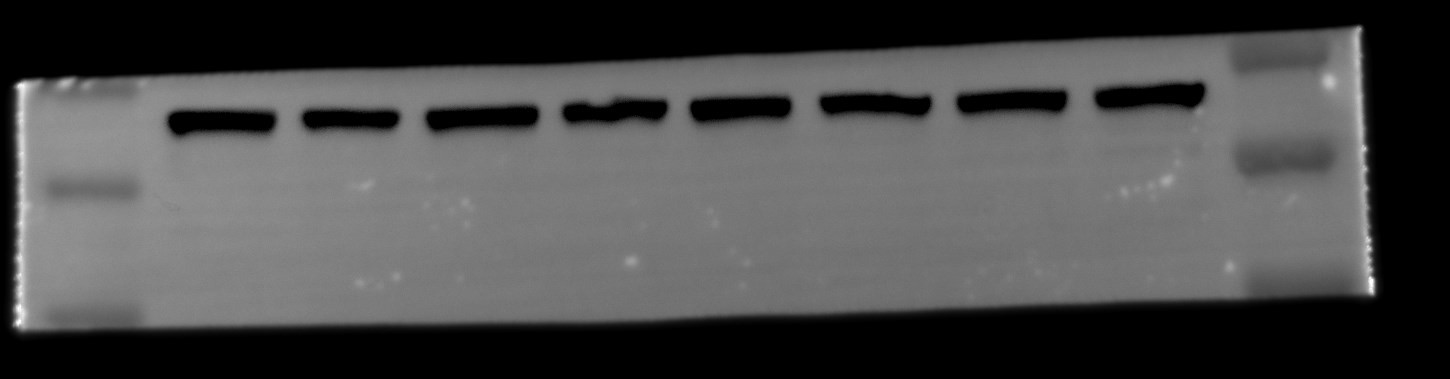

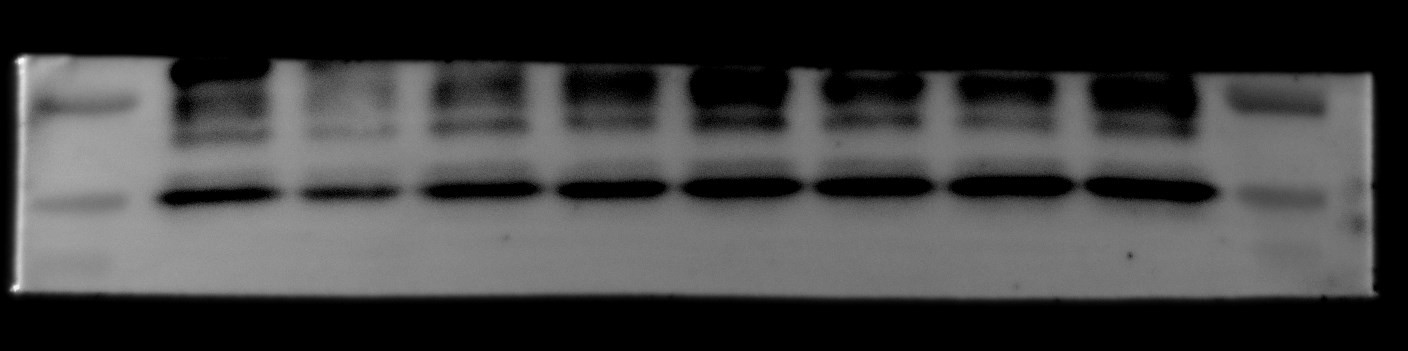


Jurkat CAR T+Nalm6 WT

0 min

15 min

19

KO

CD2

TL

19

KO

CD2

TL

**40kd**

GAPDH

**35kd**

**25kd**

**15kd**

endogenous-CD3

**10kd**


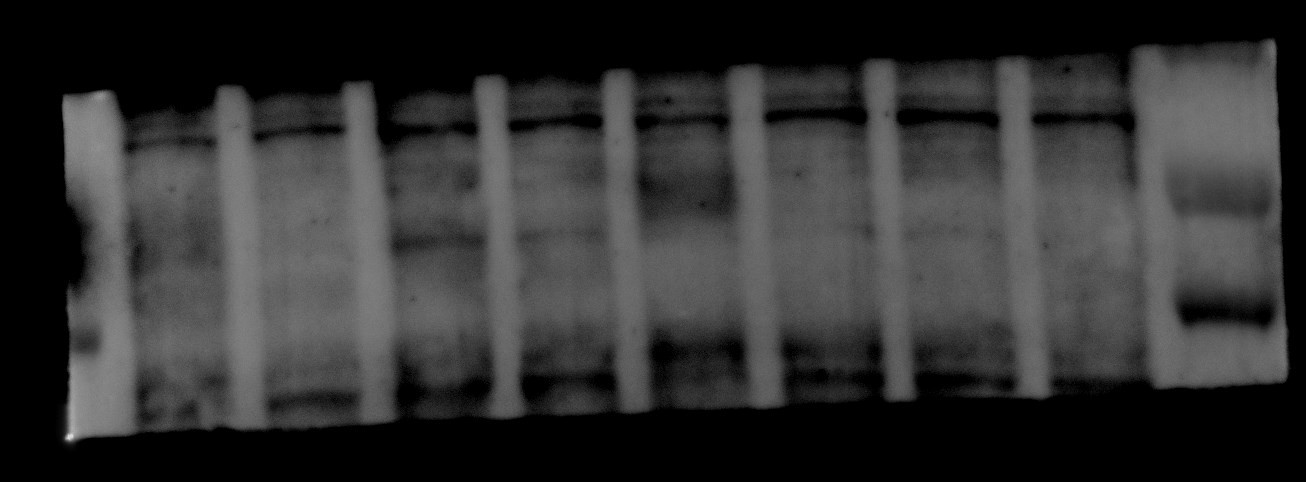


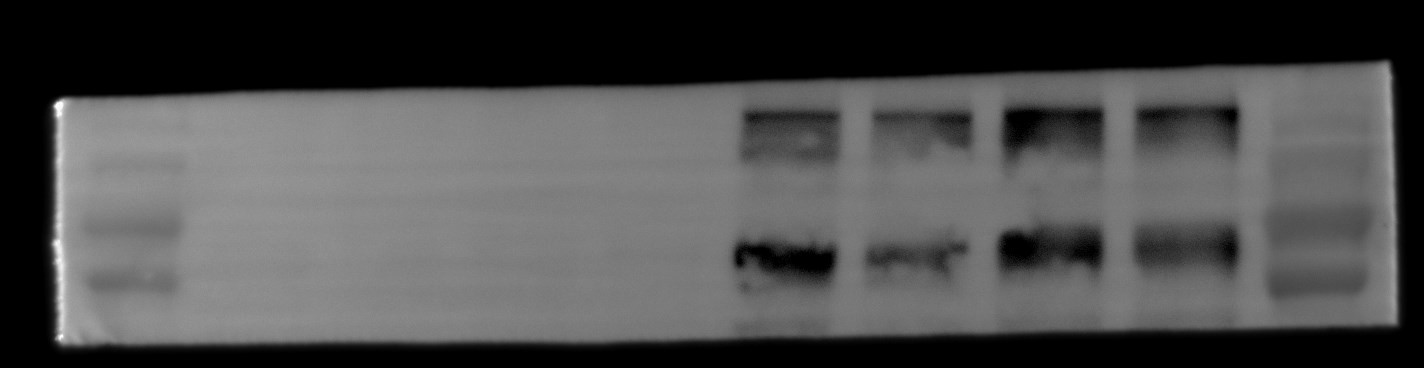


Jurkat CAR T+Nalm6 WT

15 min

0 min

19

KO

CD2

TL

19

KO

CD2

TL

**130kd 100kd 70kd**

p-CAR CD3

**100kd**

CAR CD3

**70kd 55kd**


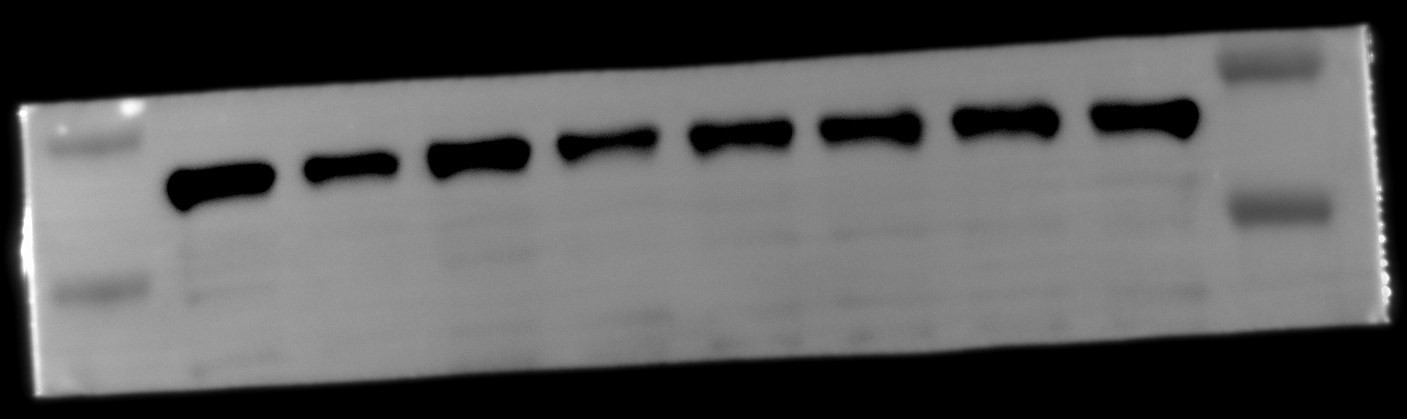

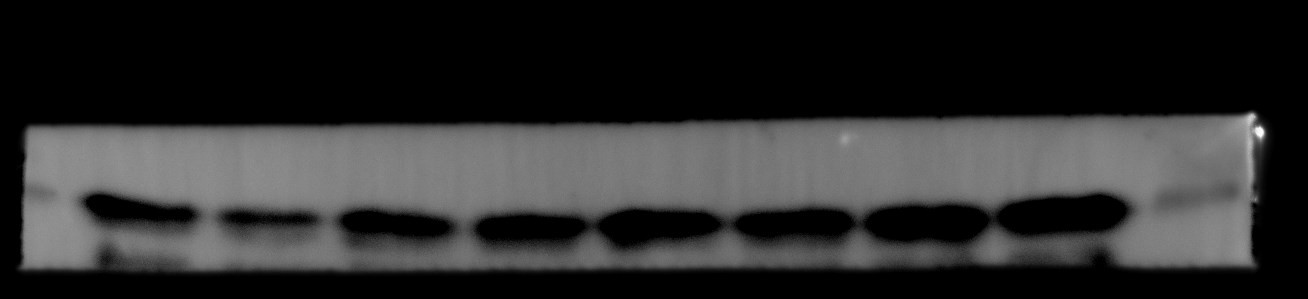


Jurkat CAR T+Nalm6 WT

0 min

15 min

19

KO

CD2

TL

19

KO

CD2

TL

**40kd**

GAPDH

**35kd**

endogenous-CD3

**10kd**


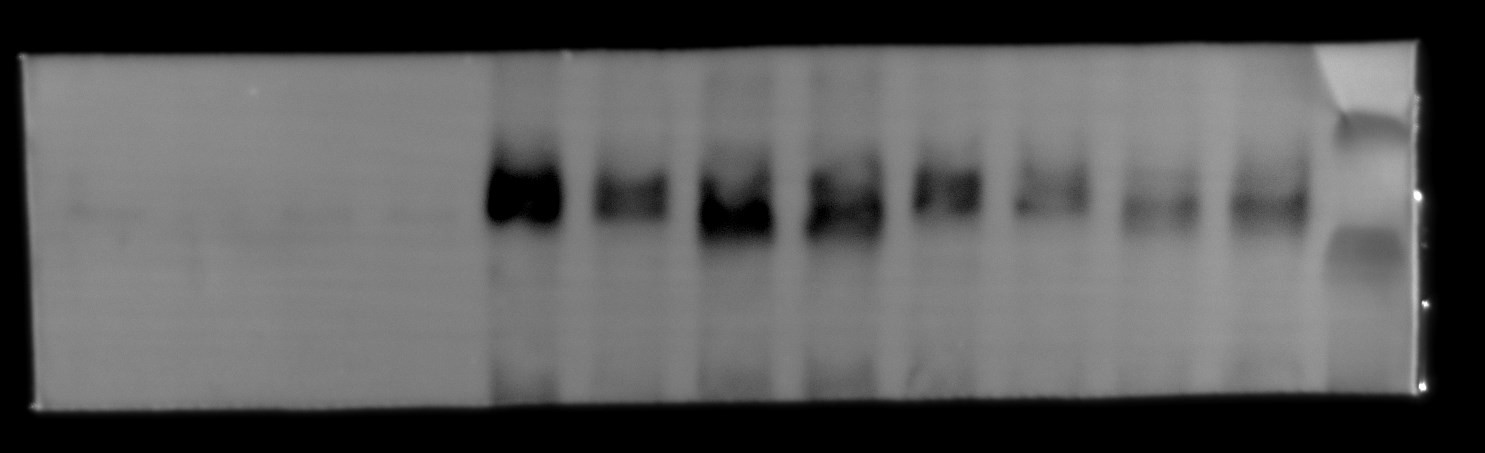

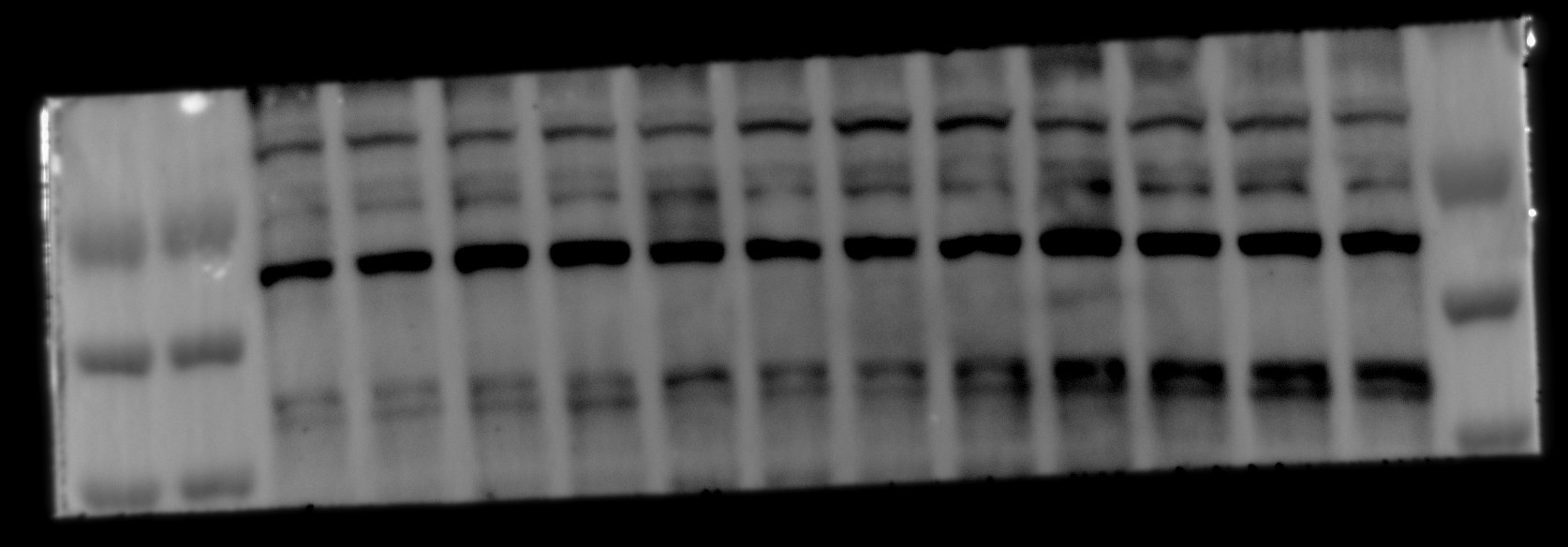


+Nalm6 WT

+Nalm6 58KO

15min

CD2

15min

CD2

0 min

TL

19

KO

CD2

19

KO

TL

19

KO

TL

**100kd**

p-CAR CD3

**700kd**

**100kd**

CAR CD3

**700kd**


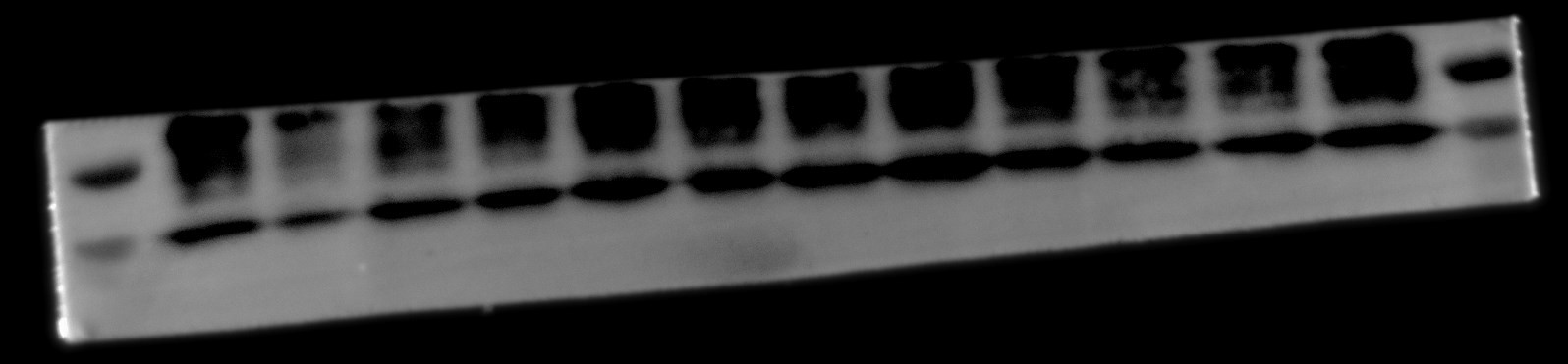

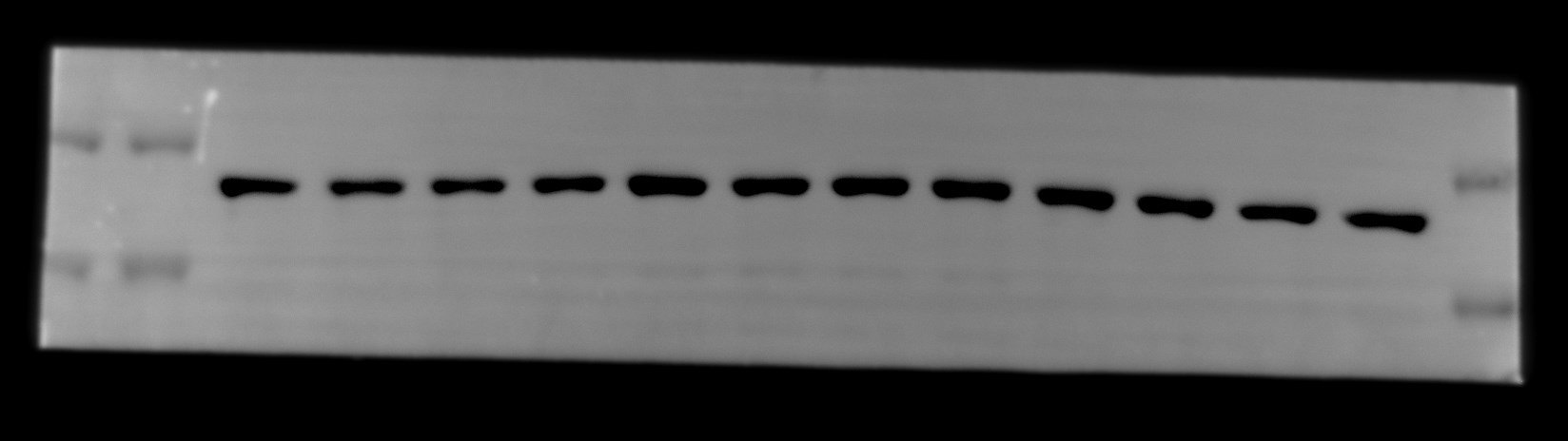


+Nalm6 WT

+Nalm6 58KO

15min

CD2

15min

CD2

0 min

TL

19

KO

CD2

19

KO

TL

19

KO

TL

**40kd**

GAPDH

**36kd**

**15kd**

**10kd**

endogenous-CD3
