# Supplementary material for: CD2 augmentation enhances CAR-T-cell efficacy via immunological synapse remodeling and T-cell exhaustion mitigation
Source: Cell Mol Immunol. 2025 Jul 4;22(8):935–48. doi: 10.1038/s41423-025-01314-6 (PMC12311108; doi:10.1038/s41423-025-01314-6)
Supplement: Supplementary file 5 — Original uncropped Western blot membranes corresponding to Figure S9B, Figure 2F and Figure 4G [file 41423_2025_1314_MOESM5_ESM.docx]

Original uncropped Western blot membranes corresponding to Figure S9B, Figure 2F and Figure 4G in the main text. Red boxes indicate the regions displayed in the main figures. Global brightness/contrast adjustments were applied as described in the Methods section.

All images are processed according to specifications without any local modifications (such as copying, liquefaction, erasure, etc.)


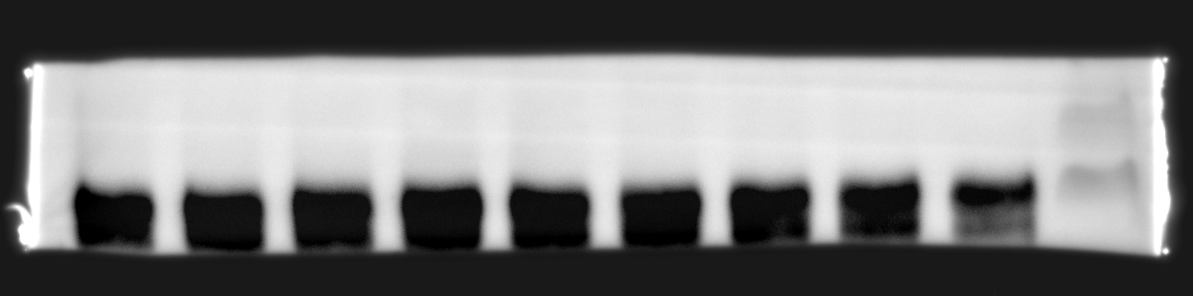

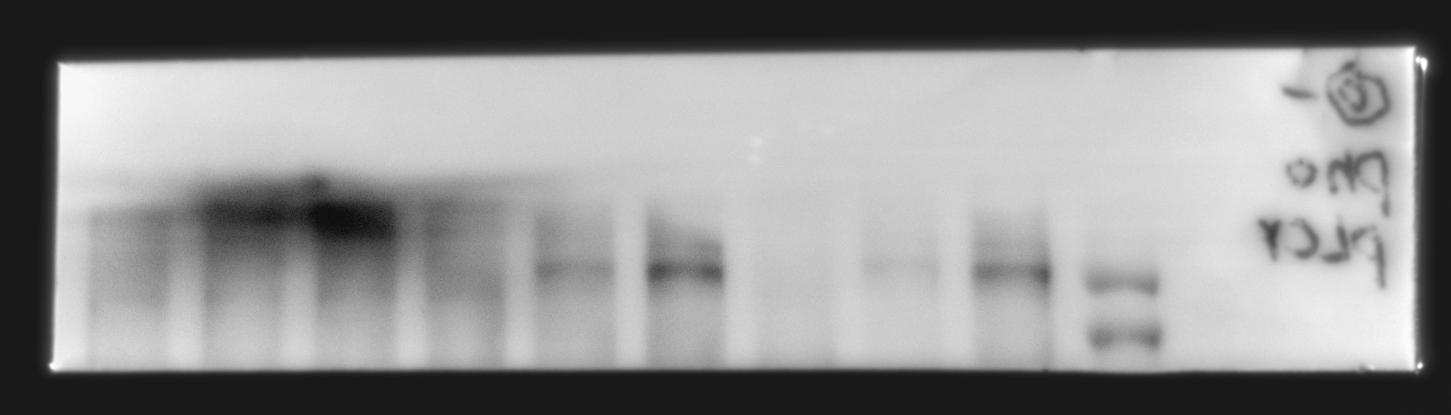


+Raji CD19^low^

PLCγ

**130kd**

**180kd**

**130kd**

15min

15min

19

WT

CD2

WT

WT

19

p-PLCγ

**180kd**

Marker

CD2

+Raji WT

CD2

19

0 min


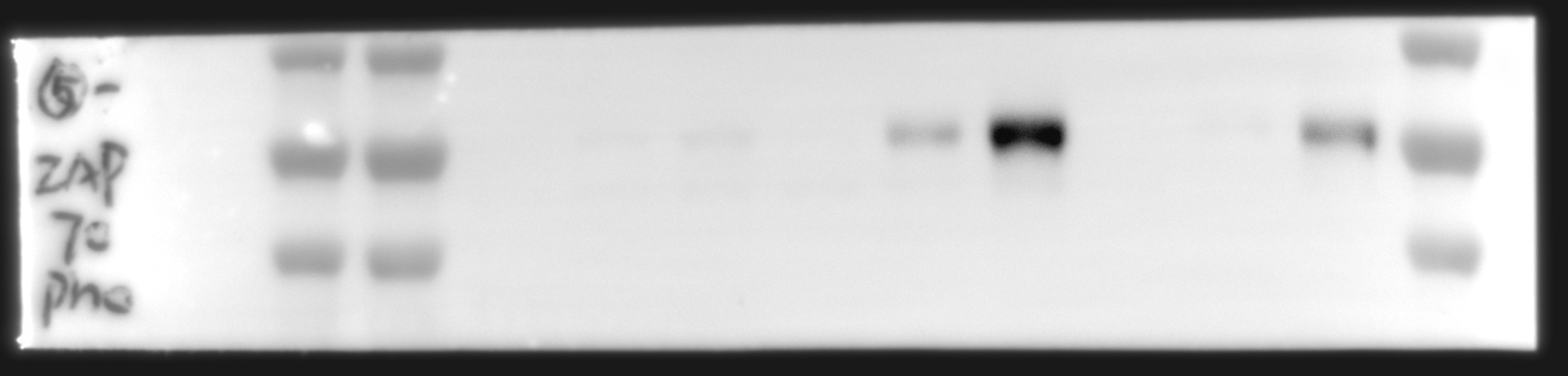

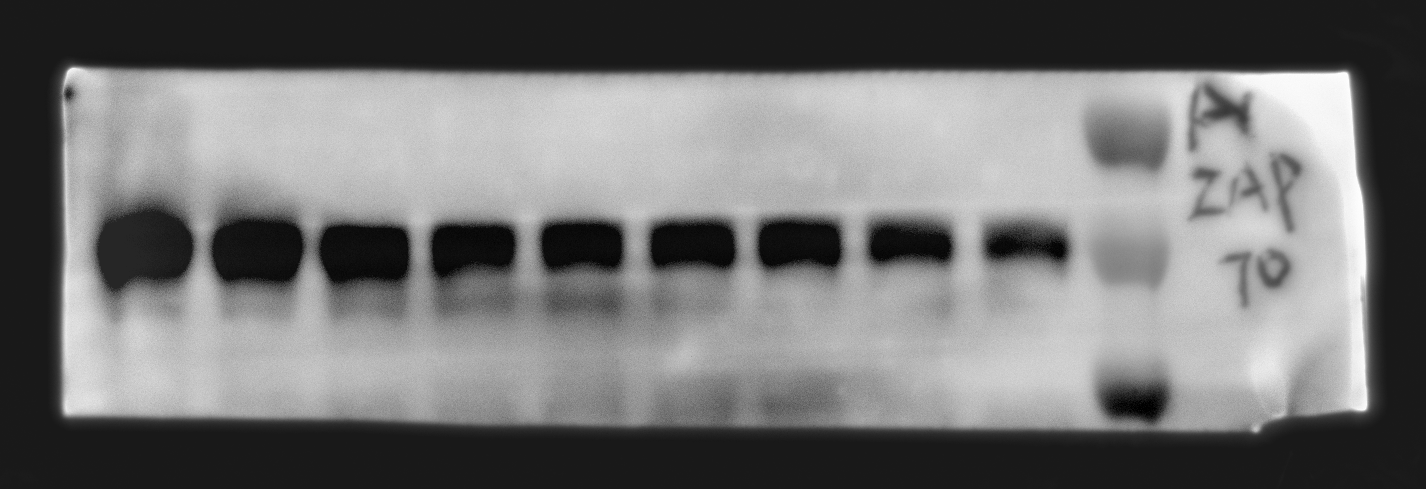
PLCγ
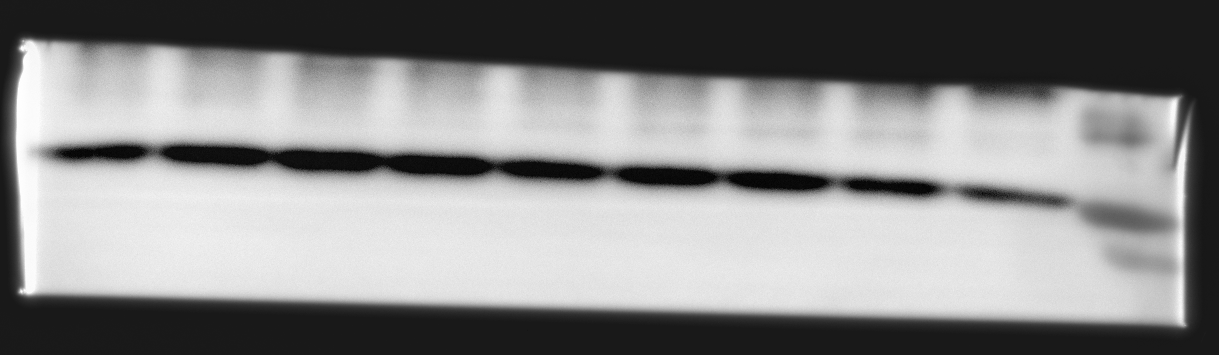

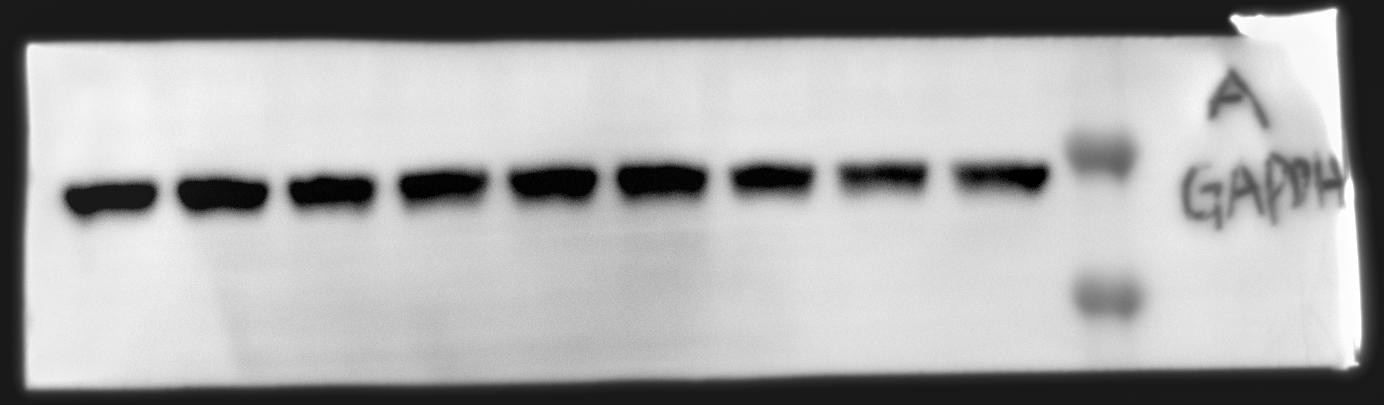


+Raji CD19^low^

ZAP70

p-ZAP70

**55kd**

**100kd**

**70kd**

**55kd**

**70kd**

**100kd**

WT

CD2

19

CD2

WT

+Raji WT

15min

0 min

15min

WT

19

19

CD2

Marker

+Raji CD19^low^

**10kd**

**15kd**

endogenous-CD3

GAPDH

**35kd**

**40kd**

15min

15min

19

WT

CD2

WT

WT

19

Marker

CD2

+Raji WT

CD2

19

0 min

PLCγ

WT

CD2

19

0 min


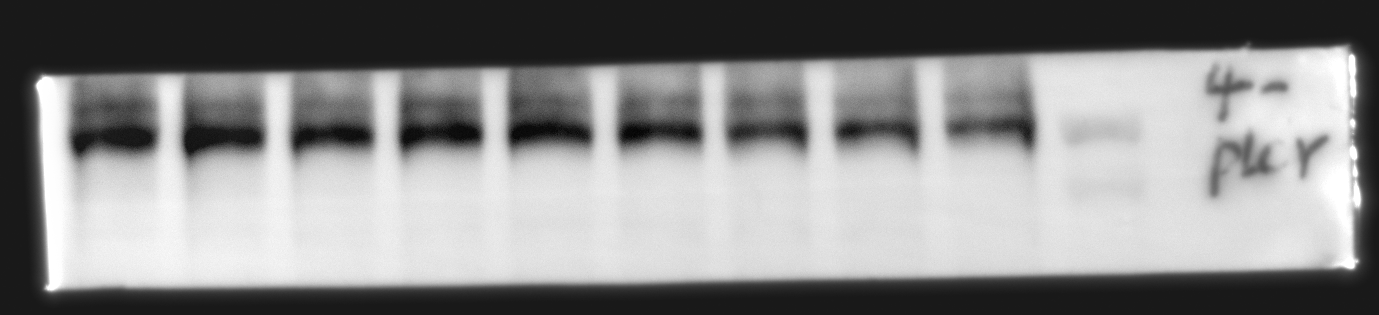

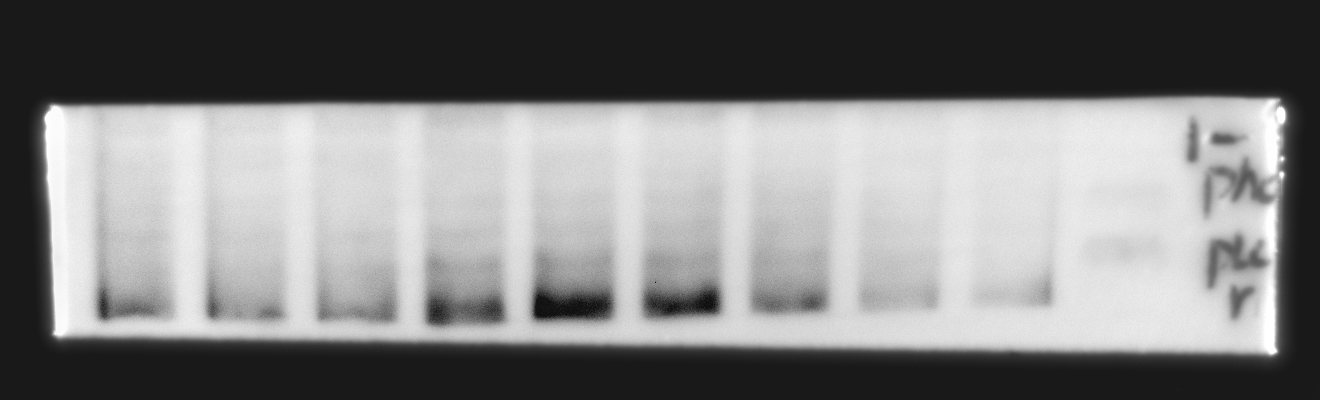


p-PLCγ

**130kd**

**180kd**

**130kd**

+K562

Marker

15min

1h

CD2

19

WT

CD2

19

WT

WT

CD2

19

0 min


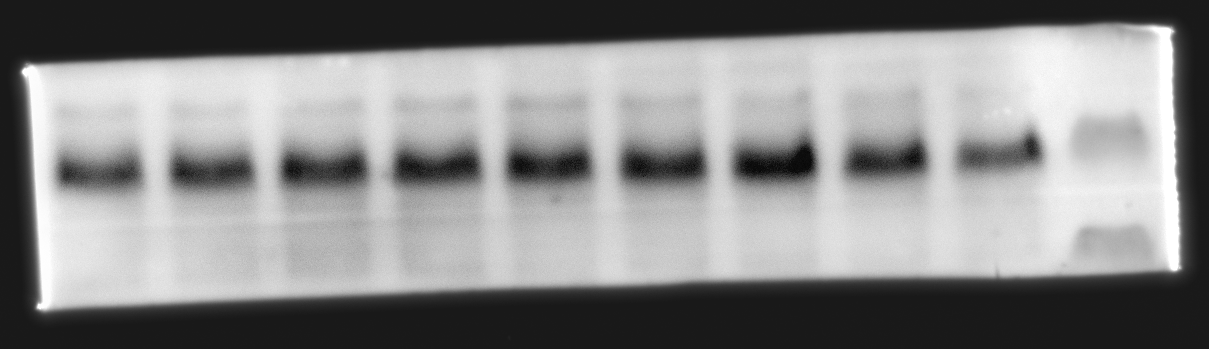

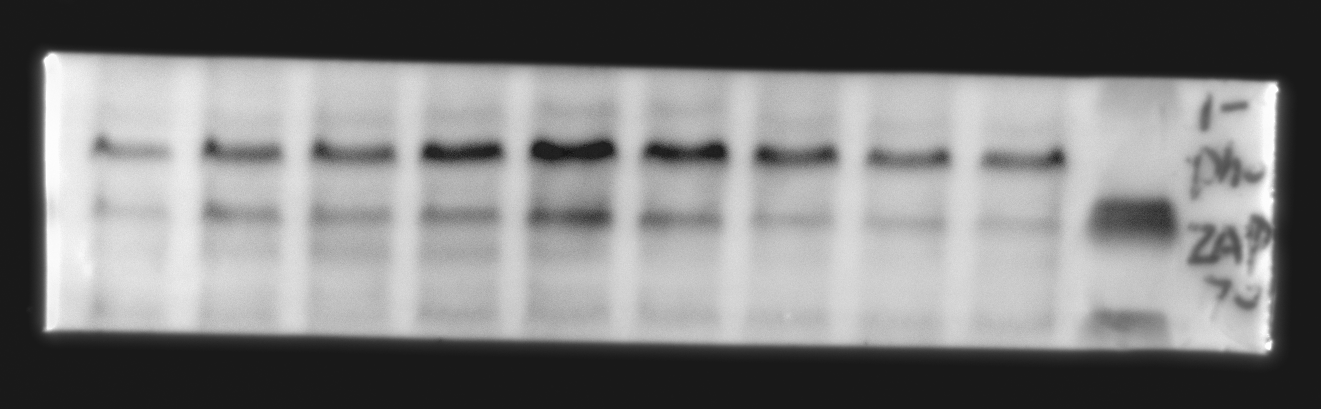


p-Zap70

Zap70

**70kd**

**70kd**

CD2

19

WT

CD2

19

WT

+K562

Marker

15min

1h

WT

CD2

19

0 min


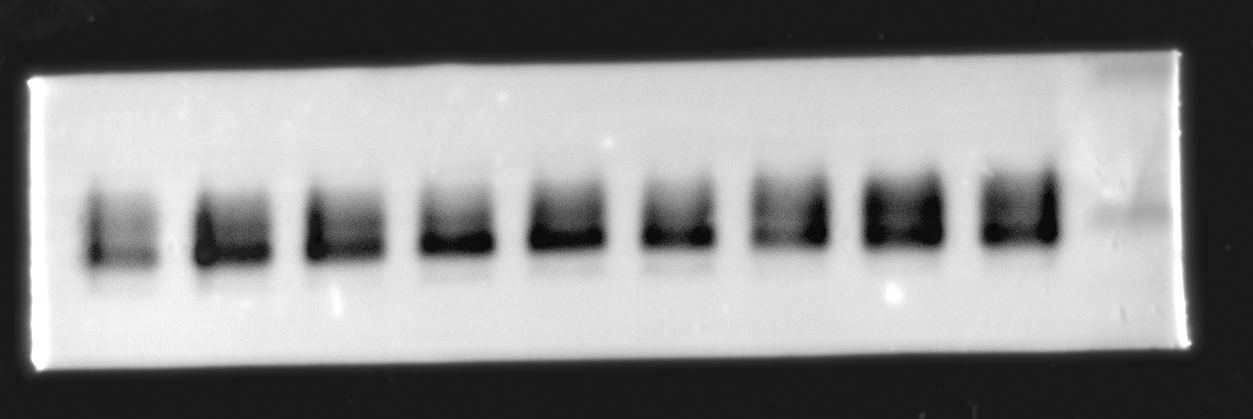

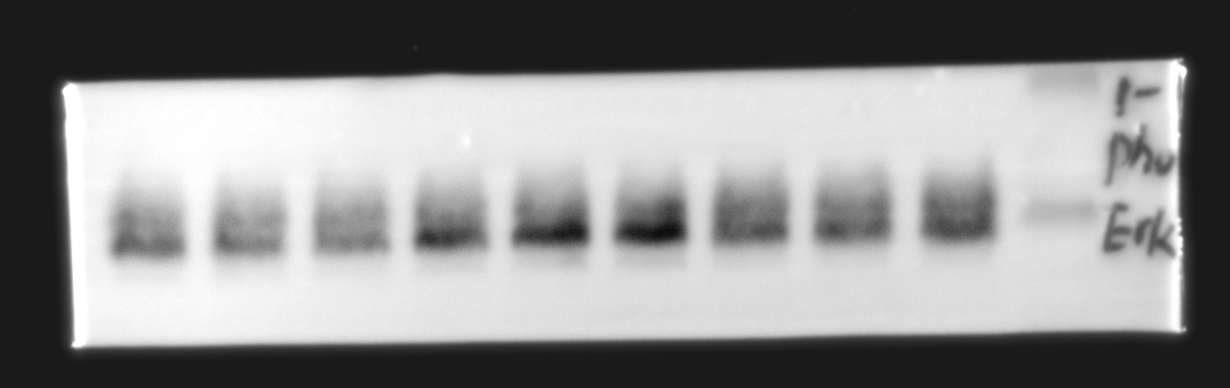


**40kd**

**40kd**

Marker

p-ERK1/2

ERK1

**70kd**

CD2

19

WT

CD2

19

WT

+K562

15min

1h

WT

CD2

19

0 min

WT

CD2

19

0 min


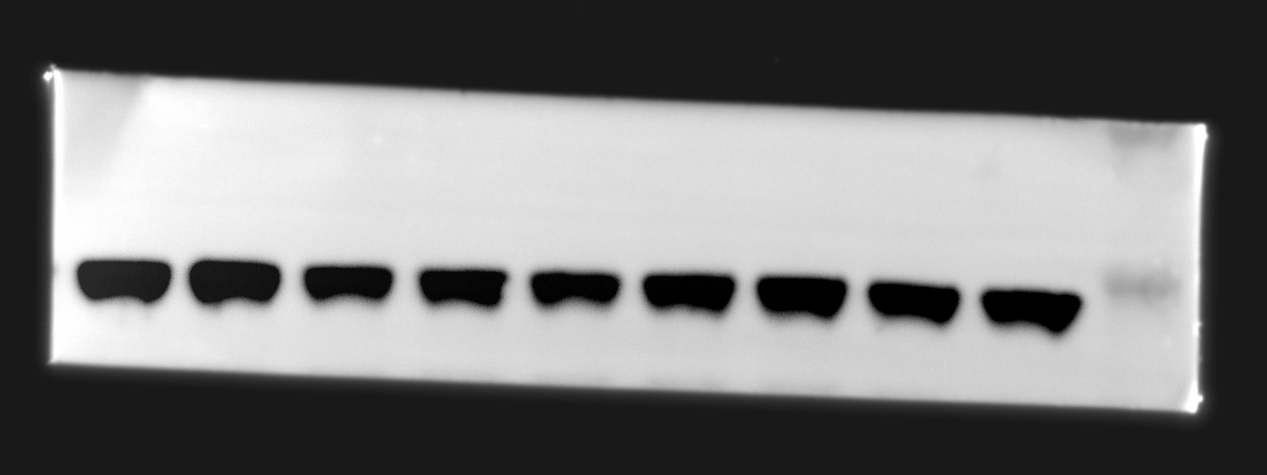


**40kd**

Marker

GAPDH

CD2

19

WT

CD2

19

WT

+K562

15min

1h


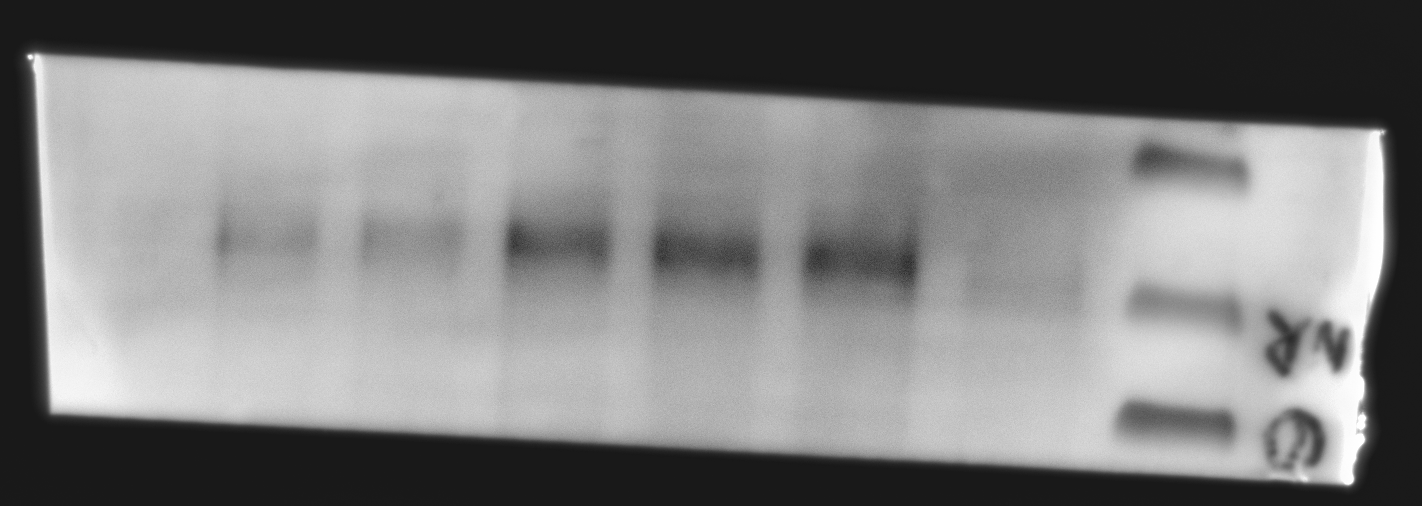

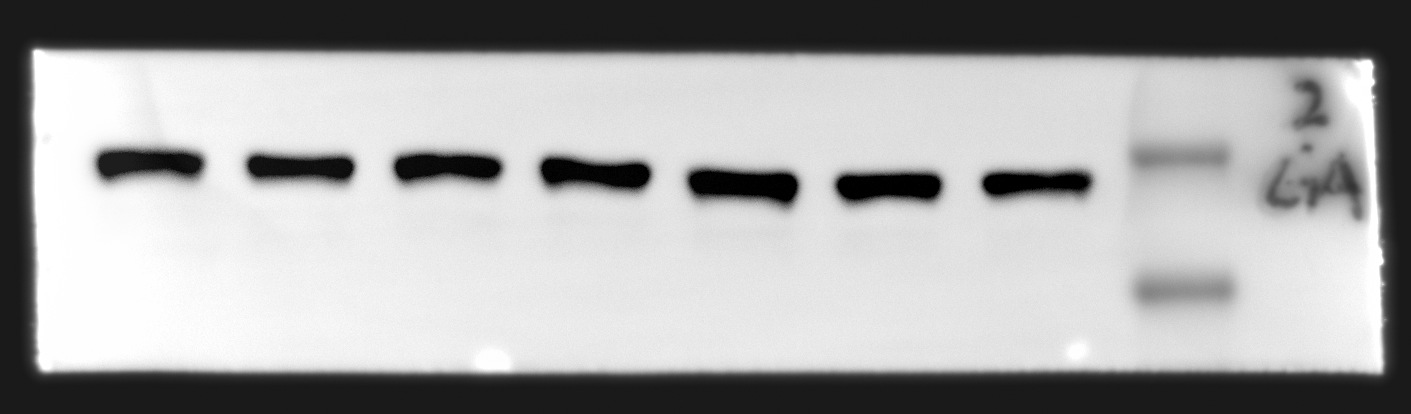


**55kd**

**40kd**

NR4A1

GAPDH

**36kd**

**70kd**

**100kd**

T

Marker

**3R**

**1R**

**0 R**

CD2

19

19

CD2

19

CD2
